# ﻿The Trichoptera of Panama XXIV. Fifteen new species and two new country records of the caddisfly genus *Neotrichia* (Trichoptera, Hydroptilidae), with a key to all known Panamanian species

**DOI:** 10.3897/zookeys.1188.111346

**Published:** 2024-01-03

**Authors:** Steven C. Harris, Brian J. Armitage, Tomás A. Ríos González

**Affiliations:** 1 Museo de Peces de Agua Dulce e Invertebrados, Universidad Autónoma de Chiriquí, David, Panama Universidad Autónoma de Chiriquí David Panama; 2 Department of Biology, Pennsylvania Western University–Clarion, Clarion, PA 16214, USA Pennsylvania Western University–Clarion Clarion United States of America; 3 Sistema Nacional de Investigación de Panamá (SNI), Panama, Panama Sistema Nacional de Investigación de Panamá Panama Panama

**Keywords:** Aquatic insects, biodiversity, freshwater, Neotropics, protected areas

## Abstract

In this paper, 15 new species of microcaddisflies in the genus *Neotrichia* Morton, 1905 (Trichoptera, Hydroptilidae) from Panama are described and illustrated: *Neotrichiaabrebotella***sp. nov.**; *Neotrichiacandela***sp. nov.**; *Neotrichiacodaza***sp. nov.**; *Neotrichiaembera***sp. nov.**; *Neotrichiaflennikeni***sp. nov.**; *Neotrichiahonda***sp. nov.**; *Neotrichialandisae***sp. nov.**; *Neotrichialenati***sp. nov.**; *Neotrichiamindyae***sp. nov.**; *Neotrichiapanamensis***sp. nov.**; *Neotrichiaparajarochita***sp. nov.**; *Neotrichiaparaxicana***sp. nov.**; *Neotrichiasnixae***sp. nov.**; *Neotrichiaspangleri***sp. nov.**; *Neotrichiaveraguasensis***sp. nov.** In addition, two new country records are presented: *Neotrichiaminutisimella* (Chambers, 1873) and *Neotrichiavibrans* Ross, 1944. Finally, the male of *N.vibrans* is re-illustrated, the female is illustrated and descriptive information given, and a key is provided to the males of all current *Neotrichia* species in Panama. There are now 45 species of *Neotrichia* and a total of 525 Trichoptera species recorded from Panama.

## ﻿Introduction

A concentrated effort during the last eight years (2015–2023) has almost doubled the known caddisfly fauna of Panama from 257 to 508 species distributed among 15 families and 56 genera (e.g., Armitage et al. 2015; Harris and Armitage 2019; Harris et al. 2023). Concomitant with the increase in species was the additional gain of two families and 11 genera (e.g., Armitage et al. 2016; Armitage and Harris 2018; Armitage et al. 2022). These increases were made possible by adoption of an integrated sampling scheme involving multiple methods (primarily UV light traps and Malaise traps in combination) employed monthly for extended periods (usually January through June) at each collection site. Our knowledge of the family Hydroptilidae has been a particular beneficiary of this approach, generating more new species to science and new country records than for all other caddisfly families combined. No microcaddisfly taxa have supported this increase more than the genus *Neotrichia* Morton, 1905.

Before 2015, only three species of *Neotrichia* were known from Panama. Since then, we have added 25 new species and new country records (Table 1). Collections from selected national parks and protected areas, as well as public and private landholdings, were made during 2017–2023 and yielded 15 new species and two new country records of *Neotrichia*. Combined with the 28 known taxa, there are now 45 species of *Neotrichia* found in Panama, with 42 of them added since 2014 (31 new species and 11 new country records). The incremental additions to Panama’s *Neotrichia* fauna are presented in Table 1, with associated literature references provided. We also re-illustrate the male of *Neotrichiavibrans* Ross, 1944 and provide descriptive text and illustrations for the female of that species. Finally, we provide a key to males of all 45 species of *Neotrichia* known from Panama.

**Table 1.** Progression of *Neotrichia* species added to Panama’s caddisfly fauna beginning in 2015*. The left column indicates the sequence numbers in The Trichoptera of Panama publication series (citations below).

Trichoptera of Panama	Year	Number of new species	Number of new country records	Species
**I**	**2015**		**1**	*Neotrichiacanixa* (Mosely, 1937)
**II**	**2015**	**2**		*Neotrichiapamelae* Harris & Armitage, 2015; *Neotrichiaparabullata* Harris & Armitage, 2015
**IV**	**2016**		**5**	*Neotrichiaesmalda* (Mosely, 1937); *Neotrichiahiaspa* (Mosely, 1937); *Neotrichiatuxtla* Bueno-Soria, 1999; *Neotrichiaunamas* Botosaneanu (in Botosaneanu and Alkins-Koo 1993); *Neotrichiaxicana* (Mosely, 1937)
**V**	**2018**	**3**		*Neotrichiaanzuelo* Armitage & Harris, 2018; *Neotrichiacollierorum* Armitage & Harris, 2018; *Neotrichiatatianae* Armitage & Harris, 2018
**VI**	**2018**	**1**		*Neotrichiaatopa* Thomson & Armitage, 2018
**X**	**2019**	**4**		*Neotrichiacarlsoni* Harris & Armitage, 2019; *Neotrichiarambala* Harris & Armitage, 2019; *Neotrichiaserrata* Harris & Armitage, 2019; *Neotrichiastarki* Harris & Armitage, 2019
	**2020**	**4**		*Neotrichiaespinosa* Armitage & Harris, 2020; *Neotrichiamichaeli* Armitage & Harris, 2020; *Neotrichiapierpointorum* Armitage & Harris, 2020; *Neotrichiayayas* Armitage & Harris, 2020
**XVII**	**2021**		**3**	*Neotrichiaamplector* Keth, 2004; *Neotrichiaarmata* Botosaneanu, 1993 (in Botosaneanu and Alkins-Koo 1993); *Neotrichiakampa* Oláh & Johanson, 2011
**XX**	**2023**	**2**		*Neotrichiamajagua* Harris, Ríos & Aguirre, 2023; *Neotrichiasolapa* Harris, Ríos & Aguirre, 2023
**XXIV**	**2023**	**15**	**2**	New species or new country records in this publication: *Neotrichiaabrebotella* sp. nov.; *Neotrichiacandela* sp. nov.; *Neotrichiacodaza* sp. nov.; *Neotrichiaembera* sp. nov.; *Neotrichiaflennikeni* sp. nov.; *Neotrichiahonda* sp. nov.; *Neotrichialandisae* sp. nov.; *Neotrichialenati* sp. nov.; *Neotrichiamindyae* sp. nov.; *Neotrichiaminutisimella* (Chambers, 1873); *Neotrichiapanamensis* sp. nov.; *Neotrichiaparajarochita* sp. nov.; *Neotrichiaparaxicana* sp. nov.; *Neotrichiasnixae* sp. nov.; *Neotrichiaspangleri* sp. nov.; *Neotrichiaveraguasensis* sp. nov.; *Neotrichiavibrans* Ross, 1944
**Totals**:		**31**	**11**	

Trichoptera of Panama publication series: **I**-Armitage et al. 2015; **II**-Harris and Armitage 2015; **IV**-Armitage et al. 2016; **V**-Armitage and Harris 2018; **VI**-Thomson and Armitage 2018; **X**-Harris and Armitage 2019; **XIV**-Armitage and Harris 2020; **XVII**-Armitage et al. 2022; **XX**-Harris et al. 2023; **XXIV**-this paper. * Prior to 2015, three species of *Neotrichia* were known from Panama: *N.flowersi* Harris, 1990; *N.malickyi* Harris (in Harris and Tiemann 1993); and *N.tauricornis* Malicky, 1980.

The Aquatic Invertebrate Research Group (**AIRG**) at the Universidad Autónoma de Chiriquí (**UNACHI**) and its Museo de Peces de Agua Dulce e Invertebrados (**MUPADI**) is currently focused on increasing our knowledge of Trichoptera (caddisflies) and Plecoptera (stoneflies) in Panama. Toward that goal, it has secured registered projects for these two orders of aquatic insects. The new taxonomic information presented in this paper are a direct result of executing these projects.

## ﻿Materials and methods

### ﻿Protected areas or private landholdings

Some of the collection sites referenced in this paper are found on private landholdings or national protected areas. These include the following study areas. They are referred to in a number of ways throughout the text. We are constrained from unifying the naming system for these because the collection label names and administrative codes for the various locations cannot be changed. Therefore, we present the Spanish names for each study area as a header followed by any abbreviations or label names used in parentheses. In the text that follows each study area, we provide the English version of the study area name, if in common usage, as additional information.

#### ﻿Reserva Privada Landis

**Figure 1.**
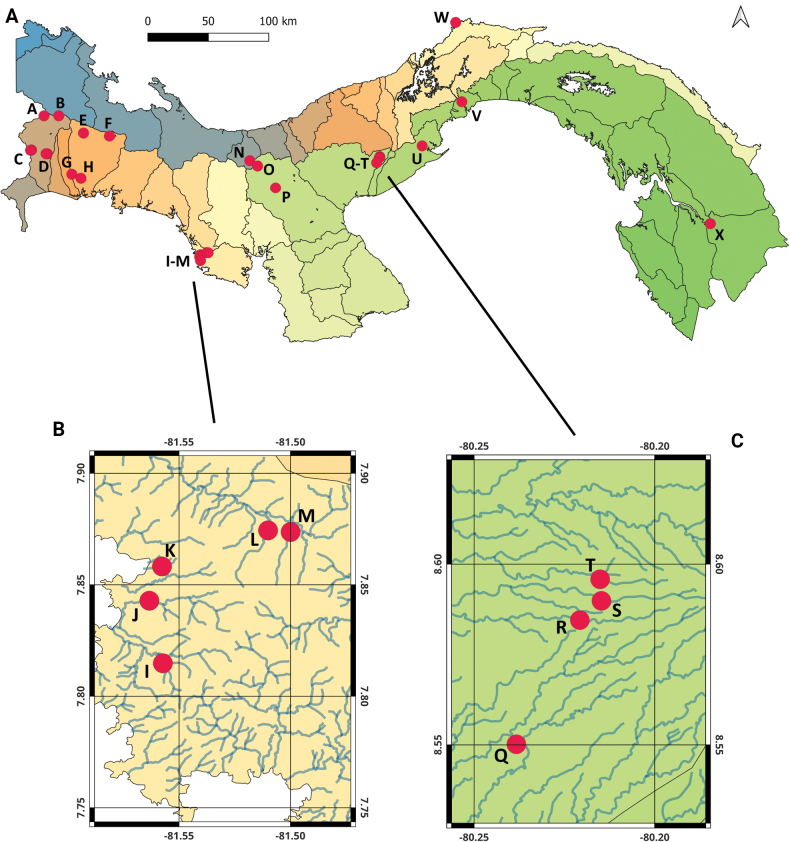
Maps **A** Panama, overlain by outlines of all 52 cuencas and showing all collection locations **B** collection locations (I–M) in the Pixvae area **C** collection locations (Q–T) in the Pajonal area. Key: A–Río Candela; B–Quebrada sin nombre; C–Quebrada sin nombre, locations 1 and 2; D–Quebrada sin nombre and Quebrada la Vuelta; E–Quebrada Grande; F–Quebrada Honda; G–Río Platanal; H–Quebrada San Cristobal; I–Quebrada Monita; J–Río Pixvae; K–Quebrada del Rosario; L–Quebrada La Mina; M–Quebrada El Rosario; N–Río Piedra de Moler; O–Quebrada Mulabá; P–Río Betegui; Q–Río Marica; R–Río Membrillo; S–Río Seren; T–Río Salado; U–Río Sajalices; V–Panama Canal; W–Quebrada sin nombre; X–Río Pirre. Note: Map colors are aesthetic, and do not impart any significance. Maps were generated using QGIS, v. 3.28.5-Firenze.

(Landis Reserve or Landis Reserva; Fig. 1A: collection location C)

Landis Reserve is a private landholding in Chiriquí Province located north of Paso Canoas, which produces plants that are used in soil stabilization efforts. The stream sampled in this study is unnamed and of first order, located near the Costa Rican border. Ultimately, it flows into the Río Chiriquí Viejo and the Pacific Ocean. The riparian corridor is primarily forested with some open areas.

#### ﻿Finca La Esperanza

(Fig. 1A: collection location D)

This is a large, private landholding in Chiriquí Province near San Andres bordered by the Río Cueta and the Río Gariché, both Pacific Ocean drainages. The immediate riparian corridor is forested, although this is surrounded by agricultural lands supporting a number of permanent crops such as cacao, plantains, and native tree species.

#### ﻿Parque International La Amistad

(PILA; Fig. 1: collection locations A and B)

The Amistad International Park is a Latin American protected area shared by Panama and Costa Rica. Including a large portion of the Cordillera de Talamanca range, it encompasses approximately 401,000 ha of upland or montane tropical forest, and is an area of high diversity and endemicity.

#### ﻿Reserva Forestal Fortuna

(Fortuna Forest Reserve; Fig. 1A: collection location F)

Part of Panama’s National System of Protected Areas, the Reserva Forestal Fortuna comprises 19,000 ha of primarily cloud forests, and an additional 500 ha of buffer zones. The 1,000 ha Fortuna Reservoir is a principal component of this protected area and is part of the Pacific drainage. The Reserva is also part of the Mesoamerican Biological Corridor, which includes the adjacent, and much larger, Bosque Protector Palo Seco (Caribbean Drainage) and Parque International La Amistad (Pacific and Caribbean drainage).

#### ﻿Parque Nacional Santa Fe

(Santa Fe NP or PNSF; Fig. 1A: collection locations N and O)

Located in the upper portion of the Santa Maria River basin in Veraguas Province, Santa Fe National Park lies near the Continental Divide and encompasses 72,636 ha. Occupying land on both the Caribbean and Pacific slopes, more than 95% of the parks area is covered with tree species which are evergreen, maintaining their leaves all year round.

#### ﻿Parque Nacional Altos de Campana

(Altos de Campana NP or PNAC; Fig. 1A: collection location U)

Established in 1966, Altos de Campana National Park in Panama Oeste Province is the oldest park in Panama. Covering 1,950 ha, the park lies on the Pacific slope of Panama and is covered, in part, by humid tropical and premontane forests.

#### ﻿Parque Nacional Darién

(Darién NP or Darién National Park; Fig. 1A: collection location X)

Darién National Park encompasses some 579,000 ha in Darién Province, adjacent to Colombia in southeastern Panama. Under formal protection since 1972, the Alto Darién Protection Forest became a national park in 1980, a World Heritage area since 1981, and, soon thereafter, a biosphere reserve by UNESCO.

All of the national parks and forest reserves indicated above are under the stewardship of the Ministerio de Ambiente de Panamá (MiAmbiente) and protected from logging and agriculture. The streams sampled under this project are 1^st^ to 3^rd^ order in size, are of good water quality, and are bordered by extensive, forested riparian corridors. The private landholdings above include some agricultural activities; however, all or almost all streams are associated with well-vegetated riparian corridors as they traverse the properties. Most of the streams are found in major watersheds (cuencas), including cuencas 102, 108, 115, 117, 132, 134, and 138, which are characterized in Cornejo et al. (2017). Cuenca 097 (Río Calovébora watershed; Caribbean drainage for Santa Fe National Park), Cuenca 104 (Río Escárrea; Pacific drainage), and Cuenca 116 (rivers between the Tabasará and the San Pablo; Pacific drainage), however, are not included in that book.

### ﻿Methods

Collections from selected national protected areas, private landholdings, and public areas were made during 2017–2023. Both Malaise and UV light traps were used for collecting aquatic insects from streams in the national parks and protected areas of Panama. Single, overnight collections were made using UV light traps (Calor and Mariano 2012). Generally, multiple-night collections were made employing Malaise traps over four-day periods. Specimens were prepared and examined following standard methods outlined in Blahnik and Holzenthal (2004). Male genitalia were soaked in 5% KOH overnight, and washed in weakly acidified alcohol prior to examination under a dissecting scope. For illustrations, specimens were slide-mounted and viewed at 250× magnification.

Morphological terminology used for male genitalia generally follows that of Marshall (1979) and classification within the Hydroptilidae follows Thomson (2023). Paired structures are described in the singular for simplicity. Although technically, segments V through X are not part of the genitalia, traditionally descriptions of segments VII through X have been included under the genitalia heading. We follow that practice here. If segments V and VI have distinct features, they are discussed under the male description. Total length of specimens provided in descriptions represents the length from the tip of the head to the tip of the forewing. Altitude values are given in meters above sea level (m a.s.l.). Maps were created using QGIS software, version 3.28.5-Firenze.

Holotypes listed in this publication are deposited in the University of Panama’s Museo de Invertebrados (**MIUP**) or MUPADI. Paratypes and other specimens are deposited in MUPADI, the University of Minnesota Insect Collection (**UMSP**), or the first author’s reference collection (**SCH**). The species listed below are in alphabetic order.

## ﻿Results

### ﻿Taxonomy—new species

#### ﻿Genus Neotrichia

Taxon classificationAnimaliaTrichopteraHydroptilidae

Morton, 1905

506FDA74-F2AE-557D-8E44-2AFFE1BA19D7

##### Remark.

The genus *Neotrichia* (Hydroptilidae, Neotrichiinae) has a New World distribution with 211 species known from North, Central, and South America and the West Indies (Armitage et al. 2022; Gama-Neto and Passos 2023; Harris et al. 2023; Thomson 2023). In the Neotropics, there are 174 species recorded (Armitage et al. 2022; Gama-Neto and Passos 2023; Harris et al. 2023; Thomson 2023). Herein we describe and illustrate 15 new species and report two new country records for Panama.

#### ﻿ Neotrichia abrebotella sp. nov.

Taxon classificationAnimaliaTrichopteraHydroptilidae

35CF34FA-F5FA-541D-979A-38D4E2532F04

https://zoobank.org/C5D20983-BF62-4562-A8D6-C4AFF10992DB

Fig. 2

##### Type locality.

**Panama: Chiriquí Province**: Cuenca 102, Renacimiento District, Reserva Privada Landis, Location 1, Quebrada sin nombre; 8.643769°N, 82.829479°W; 755 m a.s.l.

##### Type material.

***Holotype***: ♂, **Panama: Chiriquí Province**: Cuenca 102, Renacimiento District, Reserva Privada Landis, Location 1, Quebrada sin nombre; 8.643769°N, 82.829479°W; 755 m a.s.l.; 15–31.iii.2020; M. Landis leg.; Malaise trap; MUPADI-003-T-2023 (in alcohol). ***Paratypes*: Panama** • 2 ♂♂; same as holotype; except, Location 2; 8.645005°N, 82.822037°W; 575 m a.s.l.; 27.ii–6.iii.2020; M. Landis leg.; Malaise trap; MUPADI-004-T-2023 (in alcohol).

##### Diagnosis.

*Neotrichiaabrebotella* sp. nov. appears to be a member of the *N.caxima* group of Keth et al. (2015) based on the short inferior appendage with resemblance to *N.atopa* Thomson & Armitage, 2018 and *N.caxima* Mosely, 1937. It differs from *N.caxima* in the lack of sclerotized phallic processes and from *N.atopa* in the bifid appearance of the inferior appendage in lateral view and the narrow bracteole.

##### Description.

**Figure 2.**
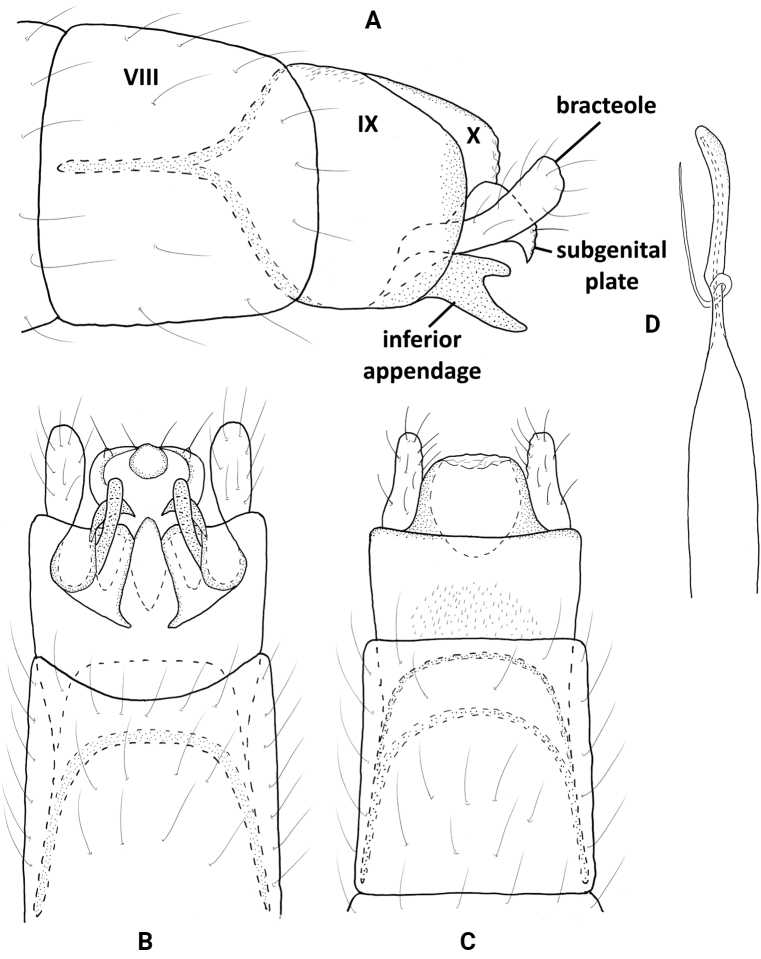
*Neotrichiaabrebotella* sp. nov., male holotype, genitalia **A** left lateral **B** ventral **C** dorsal **D** phallus, dorsal.

**Male**. Total length 1.7–1.9 mm (*n* = 3), 18 antennal segments, wings and body brown in alcohol. ***Genitalia*** (Fig. 2). Abdominal segment VIII annular. Segment IX in lateral view rounded posteriorly, narrowing dorsally, anteriorly narrowing to elongate mesal apodeme; in dorsal and ventral views quadrate, deeply incised anteriorly. Tergum X short, truncate posteriorly, tapering on lateral margins; in lateral view truncate posteriorly. Subgenital plate in lateral view wide subapically, narrowing distally to rugose, downward projecting process; in ventral view wide, truncate posteriorly with median lobe and pair of stout setae, rounded on lateral margins. Bracteole narrow basally, slightly widening distally; in dorsal and ventral views lobate. Inferior appendage short and narrow, bifid posteriorly, lower portion longer than upper; in ventral view square basally, distally bifid, dorsalmost process narrowing mesally to acute apex, ventralmost process tubular. Phallus tubular, wide basally, constricted at midlength and bearing thin paramere encircling shaft, apically thin over length.

##### Distribution.

Panama: Chiriquí Province (Reserva Privada Landis).

##### Etymology.

The species name *abrebotella* (bottle-opener) derives from Spanish, referring to the distinctive appearance of the inferior appendage. The name is a noun in the nominative singular standing in apposition.

#### ﻿ Neotrichia candela sp. nov.

Taxon classificationAnimaliaTrichopteraHydroptilidae

F289FC09-70B8-53DC-88AD-BA8481536993

https://zoobank.org/D2F4AF76-536B-4F9F-8E10-3B310C115029

Fig. 3

##### Type locality.

**Panama: Chiriquí Province**: Cuenca 102, Renacimiento District, Río Candela, Finca Félix, PILA; PSPSCB-PILA-C102-2017-021; 8.90614°N, 82.72882°W; 1799 m a.s.l.

##### Type material.

***Holotype***: ♂, **Panama: Chiriquí Province**: Cuenca 102, Renacimiento District, Río Candela, Finca Félix, PILA; PSPSCB-PILA-C102-2017-021; 8.90614°N, 82.72882°W; 1799 m a.s.l.; 1–5.ix.2017; E. Álvarez, T. Ríos, E. Pérez leg.; Malaise trap; MIUP-017-T-2023 (in alcohol).

##### Diagnosis.

*Neotrichiacandela* sp. nov. is a member of the *N.canixa* group of Keth et al. (2015) based on the posterior horns from tergum X, forked bracteoles, and the bifid inferior appendage. The new species appears to be similar to *N.bika* Oláh & Johanson, 2011 from French Guiana on the basis of the structure of the bracteoles and the horns of the tenth tergum. The new species is separated by the structure of the phallic apex, which has more elongate forking than that of *N.bika*, and by the lateral appearance of the inferior appendage and subgenital plate.

##### Description.

**Figure 3.**
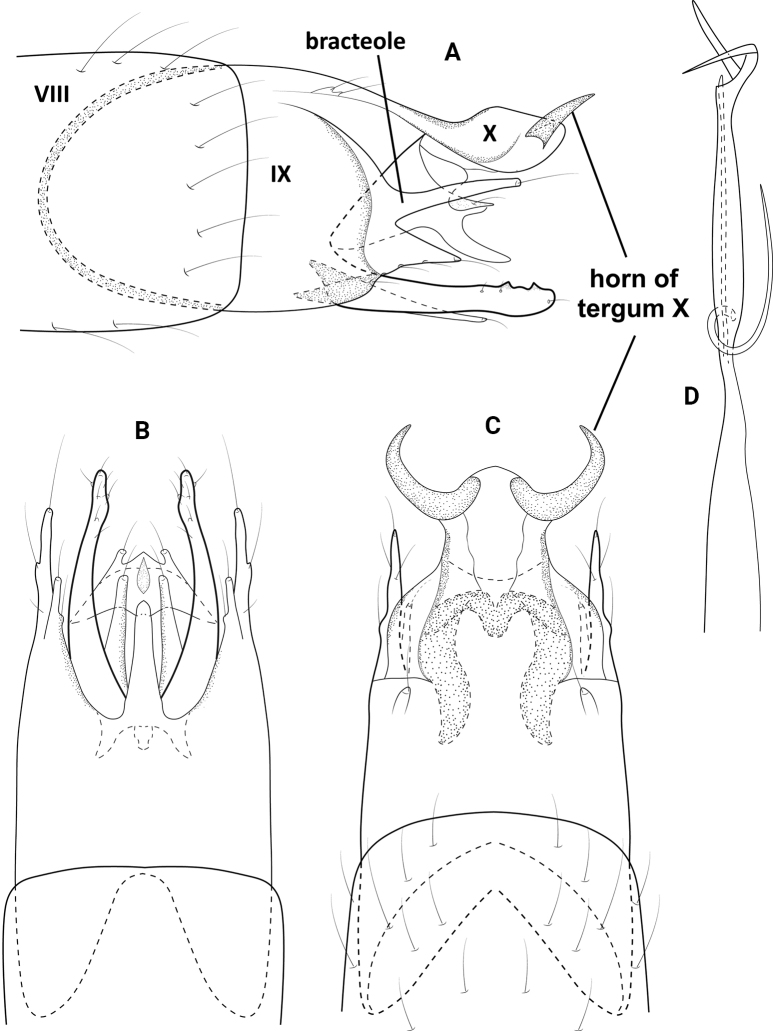
*Neotrichiacandela* sp. nov., male holotype, genitalia **A** left lateral **B** ventral **C** dorsal **D** phallus, ventral.

**Male.** Total length 1.7 mm (*n* = 1), 18 antennal segments, wings and body brown in alcohol. ***Genitalia*** (Fig. 3). Abdominal segment VIII annular. Segment IX in lateral view generally ovate, rounded anteriorly and posteriorly, fused with segment X dorsally with seta-bearing lobe posterolaterally; in ventral view anterior margin deeply incised, posterior margin with thin, elongate mesal extension; in dorsal view incomplete posteriorly. Tergum X basally fused with segment IX, wide basally, tapering posteriorly forming pair of stout, symmetrical horns; in lateral view segment X elongate, dilated distally with distal horn short and tapering to acute apex. Subgenital plate in lateral view, wide basally, tapering distally to bifid apex, upper arm short, with acute apex, lower portion elongate and crescent-shaped; in ventral view triangular-shaped, apex with pair of thin lateral lobes bearing stout setae, mesally with short distal triangle and ventral sclerotized process. Bracteole in lateral view wide anteriorly, bifid posteriorly, dorsal branch twice as long as ventral branch, each bearing terminal seta; in ventral and dorsal views both branches wide basally, tapering to rounded apices. Inferior appendage rectangular in lateral view, subapically with dorsal sclerotized points, basal process elongate and thin; in ventral view bifid, outer process slightly wider basally, curving and tapering to rounded apices, inner process fused basally and narrow over length which is ~ 1/2 of outer process, bearing elongate seta apically. Phallus tubular, constricted at midlength and bearing thin paramere encircling shaft, apex forked with elongate processes, lower of which sharply curves laterally, ejaculatory duct protruding at base.

##### Distribution.

Panama: Chiriquí Province (Parque International La Amistad).

##### Etymology.

This new species is named for the Río Candela in western Chiriquí Province where the species was collected. The name is a noun in the genitive case.

#### ﻿ Neotrichia codaza sp. nov.

Taxon classificationAnimaliaTrichopteraHydroptilidae

F5A6E68F-5EA2-5D57-BE9C-A7A0B894B83E

https://zoobank.org/F11F9E9F-D365-440D-829D-F54B295DE53E

Fig. 4

##### Type locality.

**Panama: Panama Oeste Province**: Cuenca 115, Chame District, Altos de Campana NP, Río Sajalices; PSPSCB-PNAC-C115-2018-030; 8.67625°N, 79.89748°W; 194 m a.s.l.

##### Type material.

***Holotype***: ♂, **Panama: Panama Oeste Province**: Cuenca 115, Chame District, Altos de Campana NP, Río Sajalices; PSPSCB-PNAC-C115-2018-030; 8.67625°N, 79.89748°W; 194 m a.s.l.; 29.v.2018; E. Pérez, C. Nieto, M. Molinar, T. Ríos leg.; UV light trap; MIUP-018-T-2023 (in alcohol).

##### Other material examined.

**Panama** • ♂; **Coclé Province**: Cuenca 134, Penonomé District, Río Salado, Pajonal Geosite; 8.59580°N, 80.21512°W; 323 m a.s.l.; 4.iv.2022; C. Nieto leg.; UV light trap (in alcohol) • ♂; ibid., except Río Seren; 8.58983N, 80.21476W; 332 m a.s.l. • 2 ♂♂; ibid.., except Río Membrillo; 8.58450°N, 80.22074°W; 334 m a.s.l. • 17 ♂♂; ibid., except, Río Marica, Pajonal Geosite, 8.55016°N, 80.23831°W; 316 m a.s.l. • 2 ♂♂; **Veraguas Province**: Cuenca 116, Quebrada del Rosario; 7.85826°N, 81.55764°W; 26 m a.s.l.; 20.i.2023; V. Rodriguez leg.; UV light trap • 3 ♂♂; ibid., except Cuenca 132; Río Betegui;8.36047°N, 80.99481°W; 144 m a.s.l.; 28.i.2023; V. Rodriguez leg.; UV light trap.

##### Diagnosis.

*Neotrichiacodaza* sp. nov. is a member of the *N.collata* group of Keth et al. (2015) based on the sclerites at the phallus apex and the projection from the subgenital plate. It is very similar to *N.parany* Oláh & Johanson, 2011 from Peru, based on the sharing of the distinctive pair of dark spines at the phallic apex. The new species is separated by the elongate, angled inferior appendage, and the lack of a mesal process from the posterior margin of segment IX.

##### Description.

**Figure 4.**
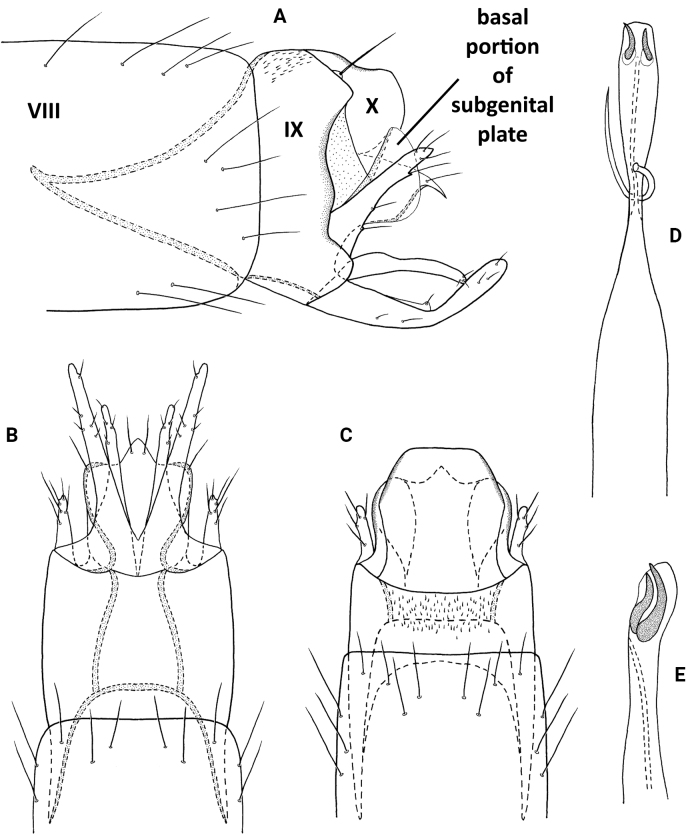
*Neotrichiacodaza* sp. nov., male holotype, genitalia **A** left lateral **B** ventral **C** dorsal **D** phallus, dorsal **E** phallus apex, left lateral.

**Male.** Total length 1.3–1.5 mm (*n* = 12), 17 antennal segments, wings and body brown in alcohol. ***Genitalia*** (Fig. 4). Abdominal segment VIII annular. Segment IX in lateral view with shallow dorsal and ventral emarginations posteriorly, anteriorly tapering to acute apex; dorsally and ventrally deeply incised anteriorly, shallow emarginations posteriorly. Segment X short and lobate in lateral view; in dorsal view apically truncate, flared laterally. Subgenital plate in lateral view, wide basally, abruptly tapering apically on venter forming acute spine, basal portion developed laterad, widening distally and flaplike; in ventral view, quadrate with apicomesal extension bearing stout setae laterad, lobate laterally. Bracteole in lateral view wide basally, tapering distally to rounded apex; in ventral and dorsal views short, tapering to rounded apex. Inferior appendage wide basally with long dorsal arm, narrowing and angled dorsad at midlength and bearing stout dorsal seta, widening distally to rounded apex; in ventral view wide basally, tapering distally to rounded apex, elongate dorsal arm wide at base, tapering distally to round apex. Phallus in dorsal view wide basally and apically, bearing short paramere encircling shaft at midlength, apex with pair of short dark spines; in lateral view apical spines are close together and slightly curving dorsally.

##### Distribution.

Panama: Panama Oeste Province (Altos de Campana National Park).

##### Etymology.

The species name *codaza* (to elbow) derives from Spanish, referring to the bent inferior appendage in lateral view. The name is a noun in the nominative singular standing in apposition.

#### ﻿ Neotrichia embera sp. nov.

Taxon classificationAnimaliaTrichopteraHydroptilidae

8AE39981-180D-519B-B40F-F1CEB0367CF3

https://zoobank.org/BB9D162B-EDD6-4CDE-A1BA-D96B38B8333D

Fig. 5

##### Type locality.

**Panama: Darién Province**: Cuenca 156, Pinogana District, Darién NP, Río Pirre, Estacion de MiAmbiente en Rancho Frio; 8.09081°N, 77.74043°W; 73 m a.s.l.

##### Type material.

***Holotype***: ♂, **Panama: Darién Province**: Cuenca 156, Pinogana District, Darién NP, Río Pirre, Estacion de MiAmbiente en Rancho Frio; 8.09081°N, 77.74043°W; 73 m a.s.l.; 9–12.ii.2018; Malaise trap; A. Thurman leg.; MUPADI-005-T-2023 (in alcohol).

##### Diagnosis.

This new species, with a pair of apical phallic spines, would appear to be another member of the *N.collata* group of Keth et al. (2015), with similarity to *N.anahua* (Mosely, 1937) from Mexico, and *N.kurtika* Oláh & Johanson, 2011 from French Guiana. Unlike these species, *N.embera* sp. nov. Harris, Armitage & Ríos has a posterolateral process from segment IX and bifid inferior appendages.

##### Description.

**Figure 5.**
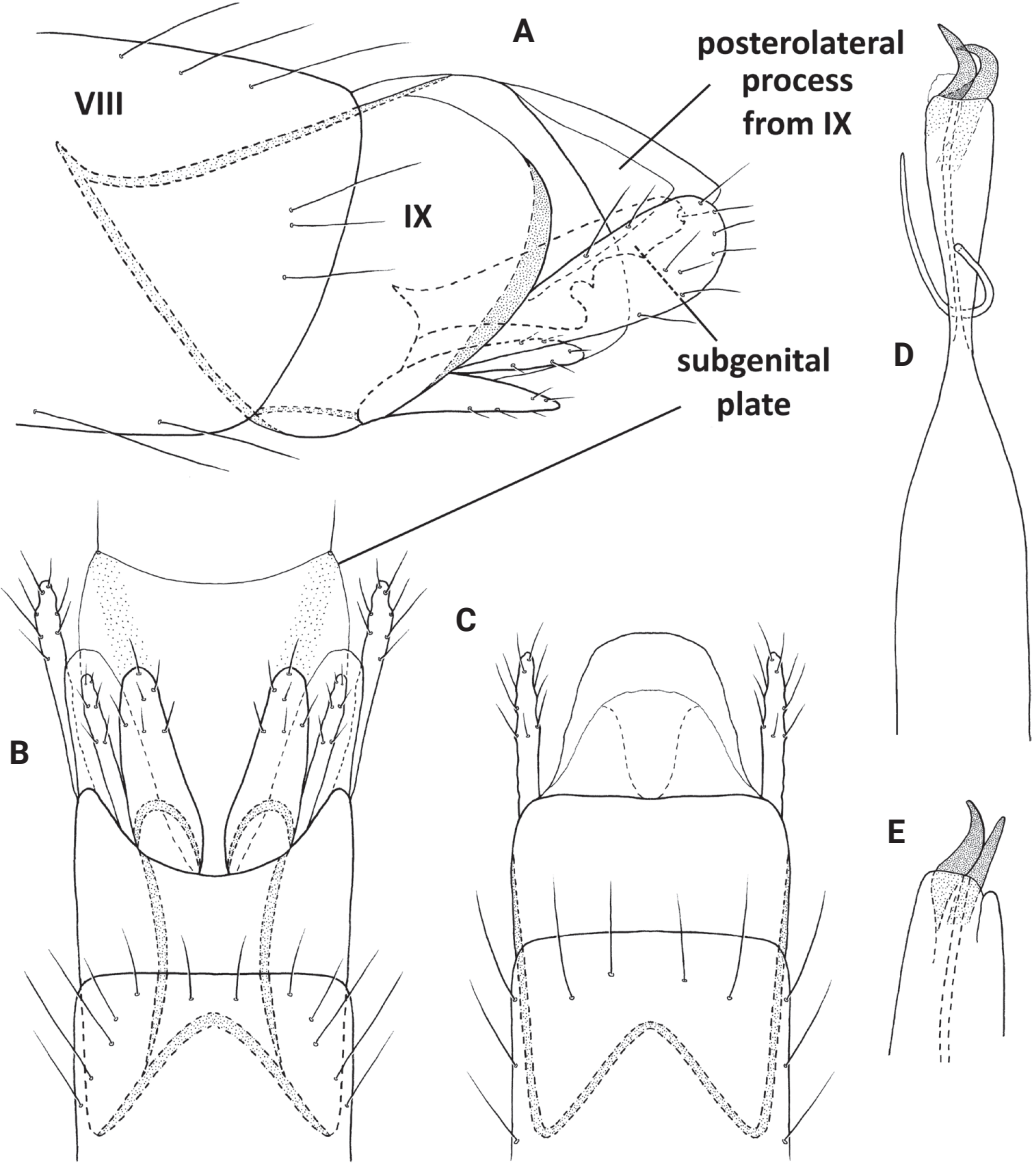
*Neotrichiaembera* sp. nov., male holotype, genitalia **A** left lateral **B** ventral **C** dorsal **D** phallus, dorsal **E** phallus apex, left lateral.

**Male.** Total length 1.2 mm (*n* = 1), 17 antennal segments, wings and body brown in alcohol. ***Genitalia*** (Fig. 5). Abdominal segment VIII annular. Segment IX in lateral view anteriorly tapering to short upturned apodeme, posteriorly with medial lobe, distally rounded with posterolateral triangular process; ventrally with anterior and posterior margins incised mesally, posteriorly with thin lateral processes; dorsally truncate posteriorly. Segment X elongate, triangular in lateral view; in dorsal view tapering distally to rounded apex. Subgenital plate in lateral view narrow basally, ventral margin sinuate with lobes and points, apex with stout seta, upper margin straight; in ventral view rectangular, widening posteriorly with stout setae on distal margins, rounded laterally. Bracteole in lateral view narrow basally, widening distally to rounded apex; in ventral and dorsal views straight, narrow over length. Inferior appendage bifid, ventral arm narrowly triangular, dorsal arm ovate and same length as lower arm; in ventral view divergent and separated, lateral arms narrow over length, inner arms slightly triangular, rounded apically. Phallus in dorsal view tubular, wide basally and distally, bearing pair of stout spines apically, short paramere encircling shaft at midlength.

##### Distribution.

Panama: Darién Province (Darién National Park).

##### Etymology.

This species is named for the indigenous Embera people of Darién Province, where the species was collected. The name is a noun in the genitive case.

#### ﻿ Neotrichia flennikeni sp. nov.

Taxon classificationAnimaliaTrichopteraHydroptilidae

E7ADEF69-8E22-51F0-858E-95E5E61E89C9

https://zoobank.org/90BFFF5E-7C6C-41C3-AF74-B0A58AB31547

Fig. 6

##### Type locality.

**Panama: Chiriquí Province**: Cuenca 108, David District, San Pablo Viejo, puente vía Interamericana antes de llegar a la entrada de Bagala, Río Platanal; 8.46416°N, 82.52030°W; 825 m a.s.l.

##### Type material.

***Holotype***: ♂, **Panama: Chiriquí Province**: Cuenca 108, David District, San Pablo Viejo, puente vía Interamericana antes de llegar a la entrada de Bagala, Río Platanal; 8.46416°N, 82.52030°W; 825 m a.s.l.; 12.iv.2021; T. Ríos, Y. Aguirre leg.; UV light trap; MUPADI-006-T-2023 (in alcohol).

##### Diagnosis.

*Neotrichiaflennikeni* sp. nov. appears to be a member of the *N.vibrans* group of Keth et al. (2015) based on the posterolateral process of segment IX, and the tapered inferior appendage. The new species appears to be similar to *N.angulata* Flint, 1983 from Uruguay which also has an elongate, tapering inferior appendage and a phallus bearing a long, stout spine. The new species is distinguished by the knob-like posterolateral processes from segment IX and by the tapering, rather than angled, appearance of the inferior appendage.

##### Description.

**Figure 6.**
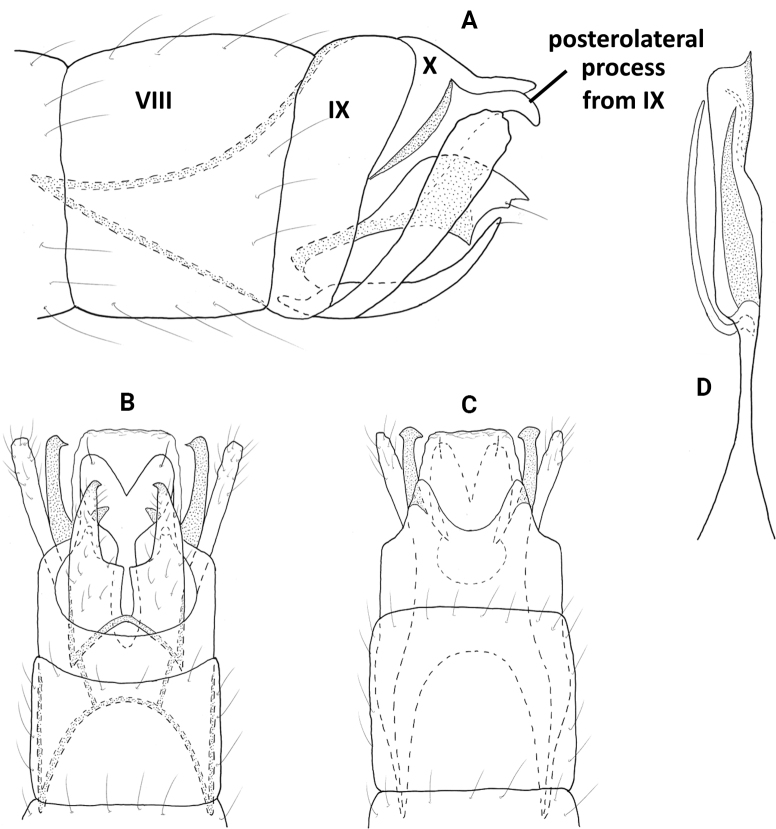
*Neotrichiaflennikeni* sp. nov., male holotype, genitalia **A** left lateral **B** ventral **C** dorsal **D** phallus dorsal.

**Male.** Total length 1.8 mm (*n* = 1), 18 antennal segments, wings and body brown in alcohol. ***Genitalia*** (Fig. 6). Abdominal segment VIII annular. Segment IX in lateral view narrow, tapering anteriorly, posteriorly with thin posterolateral process; ventrally with deep rounded posterior excision; dorsally with narrow posterior incision, posterolateral processes sclerotized and knobbed apically. Segment X narrowing posteriorly to thin shelf; in dorsal view wide basally, tapering distally to truncate apex. Subgenital plate in lateral view, narrow basally, widening distally, with acute, posteriorly projected dorsal spine, sclerotized knob posteroventrally; in ventral view narrowing basally, with pair of apical lobes each bearing stout seta. Bracteole in lateral view widening posteriorly to rounded apex; in ventral and dorsal views narrow over length. Inferior appendage in lateral view elongate and tapering, curving upward at midlength; in ventral view wide basally, sharply angled at midlength, then narrowing to acute apex which projects inward. Phallus tubular, constricted at midlength and bearing thin paramere encircling shaft, apex rectangular with elongate, stout medial spine.

##### Distribution.

Panama: Chiriquí Province (David District).

##### Etymology.

This species is named after Donald G. Flenniken of eastern Ohio, a teacher, naturalist, and part-time milkman who taught the second author (at age of 11) how to identify mammals from their skull bones/dentition and how to use taxonomic keys—a brief encounter that has led to a lifetime of taxonomic pursuits and pleasures. The name is a noun in the genitive case.

#### ﻿ Neotrichia honda sp. nov.

Taxon classificationAnimaliaTrichopteraHydroptilidae

065313C5-59DA-5FD5-AFD0-E8C9EDC0617B

https://zoobank.org/51E67AAC-F52B-4F24-BA70-C3DF7DBB5D35

Fig. 7

##### Type locality.

**Panama: Chiriquí Province**: Cuenca 108, Boquete District, Quebrada Honda, N Fortuna Dam, Fortuna Forest Reserve; 8.74985°N, 82.23885°W; 1132 m a.s.l.

##### Type material.

***Holotype***: ♂, **Panama: Chiriquí Province**: Cuenca 108, Boquete District, Quebrada Honda, N Fortuna Dam, Fortuna Forest Reserve; 8.74985°N, 82.23885°W; 1132 m a.s.l.; 18.ii.2018; B. Armitage, T. Arefina-Armitage leg.; UV light trap; MUPADI-007-T-2023 (in alcohol).

##### Diagnosis.

*Neotrichiahonda* sp. nov. does not fit well in any of the species groupings established by Keth et al. (2015). The new species has forked sclerites at the apex of the phallus and has an overall resemblance to *N.gilaensis*Keth et al., 2015 from Arizona which is in the *N.collata* group, but it is also similar to *N.yavesia* Bueno-Soria, 2010 from Mexico in the *N.vibrans* group, based on the short, rectangular inferior appendages. The new species is separated by the elongate, forked sclerites of the phallus apex, the elongate anterior apodeme of segment IX, and by the sclerotized posterolateral processes from segment IX.

##### Description.

**Figure 7.**
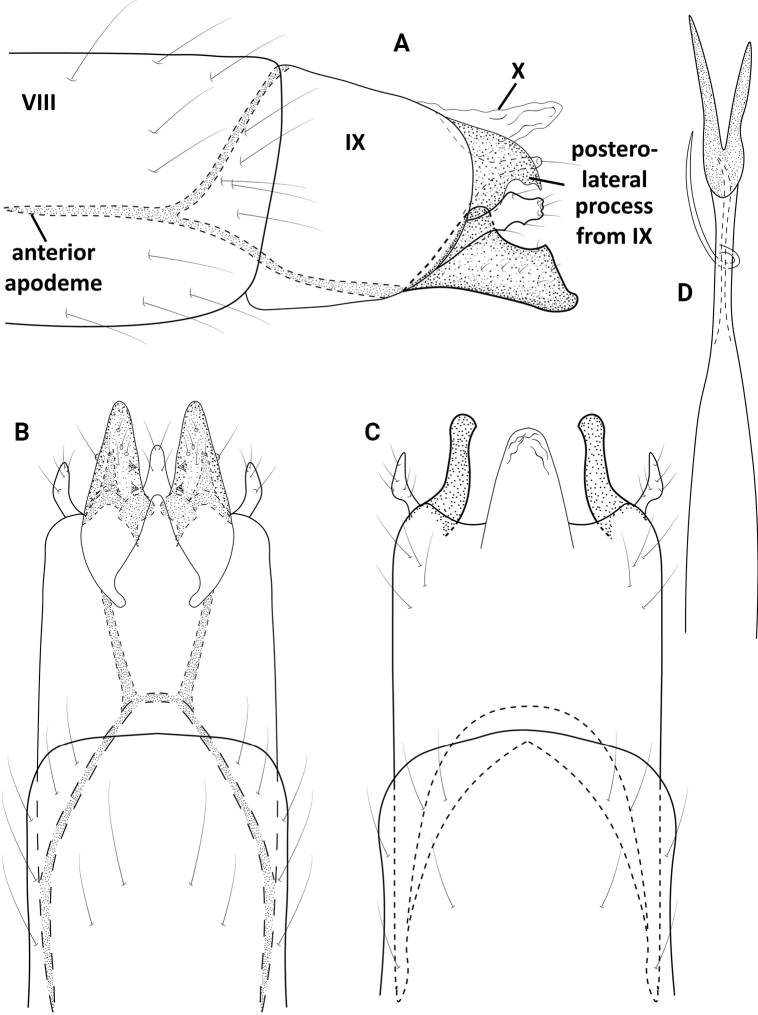
*Neotrichiahonda* sp. nov., male holotype, genitalia **A** left lateral **B** ventral **C** dorsal **D** phallus, dorsal.

**Male.** Total length 1.5 mm (*n* = 1), 18 antennal segments, wings and body brown in alcohol. ***Genitalia*** (Fig. 7). Abdominal segment VIII annular. Segment IX in lateral view anteriorly tapering to elongate apodeme, posteriorly rounded with posterolateral process, which is wide basally, tapering distally to an acute downward turning hook; dorsally and ventrally deeply incised anteriorly, ventrally with deep lateral incisions posteriorly, creating an elongate mesal, triangular lobe; dorsally with posterolateral processes narrow over length, curving slightly inward apically. Segment X elongate and thin in lateral view; in dorsal view wide basally and fused with segment IX, tapering to rounded apex. Subgenital plate in lateral view, wide basally, gradually tapering apically to rounded apex bearing elongate seta; in ventral view narrowly triangular, pair of stout setae apically. Bracteole in lateral view wide basally, then narrowing distally to truncate apex; in ventral and dorsal views narrow over length and curving mesally. Inferior appendage sclerotized and rectangular, wide basally, narrowing at midlength, then abruptly tapering to rounded apex; in ventral view triangular, rounded apically, basally with finger-like lobe. Phallus in dorsal view wide basally, bearing short paramere encircling shaft at midlength, apex divided into pair of elongate, sclerotized rods, which are nearly equal in length.

##### Distribution.

Panama: Chiriquí Province (Fortuna Forest Reserve).

##### Etymology.

This species is named for Quebrada Honda, a tributary of Río Chiriquí and Fortuna Reservoir in northeast Chiriquí Province, where the species was collected. The name is a noun in the genitive case.

#### ﻿ Neotrichia landisae sp. nov.

Taxon classificationAnimaliaTrichopteraHydroptilidae

B7F6361A-331B-5229-9C23-46B1F02ECBC0

https://zoobank.org/458EB8A1-9CD5-45DC-9A35-7F385BEC50B4

Fig. 8

##### Type locality.

**Panama: Chiriquí Province**: Cuenca 102, Renacimiento District, Reserva Privada Landis, Quebrada sin nombre, Location 2; 8.645005°N, 82.822037°W; 575 m a.s.l.

##### Type material.

***Holotype***: ♂, **Panama: Chiriquí Province**: Cuenca 102, Renacimiento District, Reserva Privada Landis, Quebrada sin nombre, Location 2; 8.645005°N, 82.822037°W; 575 m a.s.l.; 30.iv.2020; M. Landis leg.; Malaise trap; MUPADI-008-T-2023 (in alcohol). ***Paratypes*: Panama** • 9 ♂♂; ibid., except 30.v.2020; MUPADI-009-T-2023 (in alcohol).

##### Other material examined.

**Panama** • 2 ♂♂; ibid., except 27.ii–15.iii.2020 • 1 ♂; ibid., except 15–31.iii.2020 • 2 ♂♂; ibid., except Location 1, 8.643769°N, 82.829479°W; 755 m a.s.l.; 27.ii–10.iii.2020; M. Landis leg.; Malaise trap • 1 ♂; **Chiriquí Province**: Bugaba District, Cuenca 102, nr San Andres, Finca La Esperanza, Quebrada La Vuelta; 8.61710°N, 82.70415°W; 492 m a.s.l.; 3–20.i.2022; T. Ríos, Y. Aguirre leg.; Malaise trap • 2 ♂♂; ibid., except 21.i–3.ii.2022 • 3 ♂♂; ibid., except 6–20.iii.2022 • 2 ♂♂; ibid., except 8–22.iv.2022 • 2 ♂♂; ibid., except 8–21.v.2022 • 2 ♂♂; ibid., except Quebrada sin nombre; 8.61765°N, 82.71330°W; 540 m a.s.l.; 21.i–3.ii.2022.

##### Diagnosis.

*Neotrichialandisae* sp. nov. belongs to a cluster of similar Panamanian species with characteristic elongate posterolateral processes from abdominal segment IX, including *N.tatianae* Armitage & Harris, 2018 and *N.yayas* Armitage & Harris, 2020. The new species is separated from these species by the linear posterolateral processes from segment IX, the truncate inferior appendage, and the narrow subgenital plate in ventral view.

##### Description.

**Figure 8.**
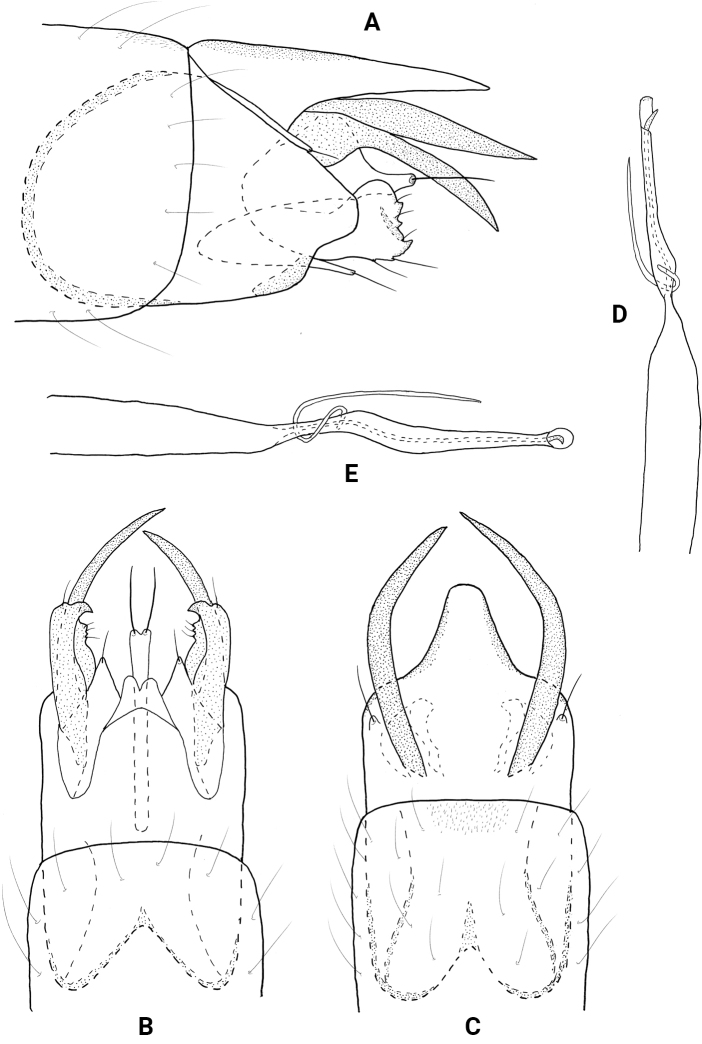
*Neotrichialandisae* sp. nov., male holotype, genitalia **A** left lateral **B** ventral **C** dorsal **D** phallus, dorsal **E** phallus, lateral.

**Male.** Total length 1.5–1.7 mm (*n* = 15), 18 antennal segments, wings and body brown in alcohol. ***Genitalia*** (Fig. 8). Abdominal segment VIII annular. Segment IX in lateral view anteriorly rounded, wide posteroventrally, narrowing dorsally, pair of elongate, thin posterolateral processes, which narrow distally; in dorsal view fused posteriorly with segment X, pair of small setaceous lobes laterally, posterolateral processes sclerotized and elongate, but dissimilar in length, anteriorly with narrow mesal incision; in ventral view with deep lateral incisions. Tergum X basally fused with segment IX, posteriorly elongate, tapering to rounded mesal lobe; in lateral view wide basally, tapering distally to acute apex. Subgenital plate in lateral view wide basally, tapering distally to narrow shelf bearing stout seta; in ventral view narrow over length, pair of setae apically. Bracteole absent or represented by thin, elongate seta-bearing process on dorsal margin of segment IX. Inferior appendage short, wide basally, widening subapically, spinose and rugose on distal margin, thin ventral process basally; in ventral view elongate and narrow, apical point turning inward, spinose subapically on mesal margin, base of inferior appendage and processes from segment IX superimposed, short triangular mesal process bearing stout seta apically. Phallus tubular, constricted at midlength and bearing thin paramere encircling shaft, apically rounded with ejaculatory duct protruding.

##### Distribution.

Panama: Chiriquí Province (Reserva Privada Landis, Finca La Esperanza).

##### Etymology.

This species is named for Senora Marietta Isabel Landis, owner of Reserva Privada Landis, who managed all collection events. The name is a noun in the genitive case.

#### ﻿ Neotrichia lenati sp. nov.

Taxon classificationAnimaliaTrichopteraHydroptilidae

6894880E-625E-5612-A2D0-5A7BB8EA8BE5

https://zoobank.org/9068A45C-197D-419C-8415-CDE94274F387

Fig. 9

##### Type locality.

**Panama: Chiriquí Province**: Cuenca102, Renacimiento District, Reserva Privada Landis, Location 1, Quebrada sin nombre; 8.643769°N, 82.82979°W; 755 m a.s.l.

##### Type material.

***Holotype***: ♂, **Panama: Chiriquí Province**: Cuenca102, Renacimiento District, Reserva Privada Landis, Location 1, Quebrada sin nombre; 8.643769°N, 82.82979°W; 755 m a.s.l.; 5–30.xii.2022; M. Landis leg.; Malaise trap; MUPADI-010-T-2023 (in alcohol).

##### Diagnosis.

*Neotrichialenati* sp. nov. appears to be a member of the *N.okopa* group of Keth et al. (2015) based on the tubular phallus which lacks sclerotized processes, the posterolateral process from segment IX, and the thin tapering inferior appendage. It is, perhaps, most similar to *N.connori*Keth et al., 2015 from Mexico and *N.teutonia* Flint, 1983 from Brazil, which also have tapering inferior appendages and a posterolateral process from segment IX. The new species is separated by the presence of a basal process from the inferior appendage, narrow bracteole, and the ventral location and shape of the posterolateral process from segment IX.

##### Description.

**Figure 9.**
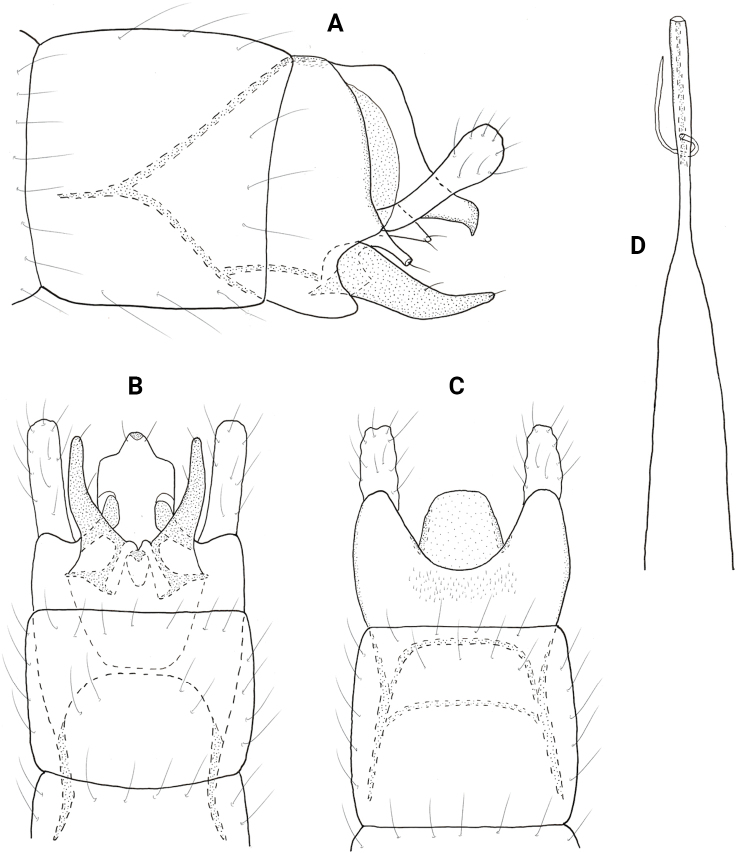
*Neotrichialenati* sp. nov., male holotype, genitalia **A** left lateral **B** ventral **C** dorsal **D** phallus dorsal.

**Male.** Total length 1.4 mm (*n* = 1), 18 antennal segment, wings and body brown in alcohol. ***Genitalia*** (Fig. 9). Abdominal segment VIII annular. Segment IX in lateral view narrow, tapering anteriorly to mesal apodeme, truncate posteriorly, with thin process posterolaterally, tapering ventrad and incised on posterior margin; in ventral view narrow, sinuate posteriorly; in dorsal view deeply incised mesally, forming rounded lateral lobes. Segment X seemingly fused with segment IX in lateral view, tapering downward to posterior projecting shelf with pointed tip; in dorsal view short, rounded distally, with truncate apex and sclerotized margins. Subgenital plate triangular in lateral view; in ventral view quadrate, lateral apices indented with stout seta, small mesal downturned lobe. Bracteole narrow in lateral view, slightly widening distally; in ventral and dorsal views narrow over length. Inferior appendage sclerotized, tapering distally and slightly curving upward at midlength, dorsobasally with thin process tipped with stout seta; in ventral view tapering distally to rounded apex, knob-like basal process on inner margin. Phallus tubular, constricted near midlength and bearing thin paramere encircling shaft, posteriorly thin and rectangular, ejaculatory duct lightly sclerotized.

##### Distribution.

Panama: Chiriquí Province (Reserva Privada Landis).

##### Etymology.

This species is named in memory of David R. Lenat of Raleigh, North Carolina, our friend and colleague, who was an avid researcher in taxonomy and aquatic bioassessment, and who made numerous important contributions to our knowledge of chironomids, stoneflies, caddisflies, mayflies, and many other groups of aquatic insects. The name is a noun in the genitive case.

#### ﻿ Neotrichia mindyae sp. nov.

Taxon classificationAnimaliaTrichopteraHydroptilidae

35DF6EFF-01C2-59B1-AFA7-928043A633D0

https://zoobank.org/7F7659D8-B935-4C3E-82CF-135B85223928

Fig. 10

##### Type locality.

**Panama: Darién Province**: Cuenca 156, Pinogana District, Darién NP, Río Pirre, Estacion de MiAmbiente en Rancho Frio; 8.09081°N, 77.74043°W; 73 m a.s.l.

##### Type material.

***Holotype***: ♂, **Panama: Darién Province**: Cuenca 156, Pinogana District, Darién NP, Río Pirre, Estacion de MiAmbiente en Rancho Frio; 8.09081°N, 77.74043°W; 73 m a.s.l.; 9–12.ii.2018; A. Thurman leg.; Malaise trap; MUPADI-011-T-2023 (in alcohol).

##### Diagnosis.

*Neotrichiamindyae* sp. nov. is another member of the *N.canixa* group of Keth et al. (2015) based on the posterior horns from tergum X, forked bracteoles, and the bifid inferior appendage. The new species appears to be most similar to *N.maya* Harris & Flint, 2016 from Belize on the basis of the structure of the bracteole and the horns of tergum X. The new species is separated by the structure of the phallic apex, which while forked, has the one process extremely long, similar to that of *N.maria* Bueno-Soria & Hamilton, 1986 from Mexico, and by the unequal forking of the bracteole, which are uniformly forked in *N.maria*.

##### Description.

**Figure 10.**
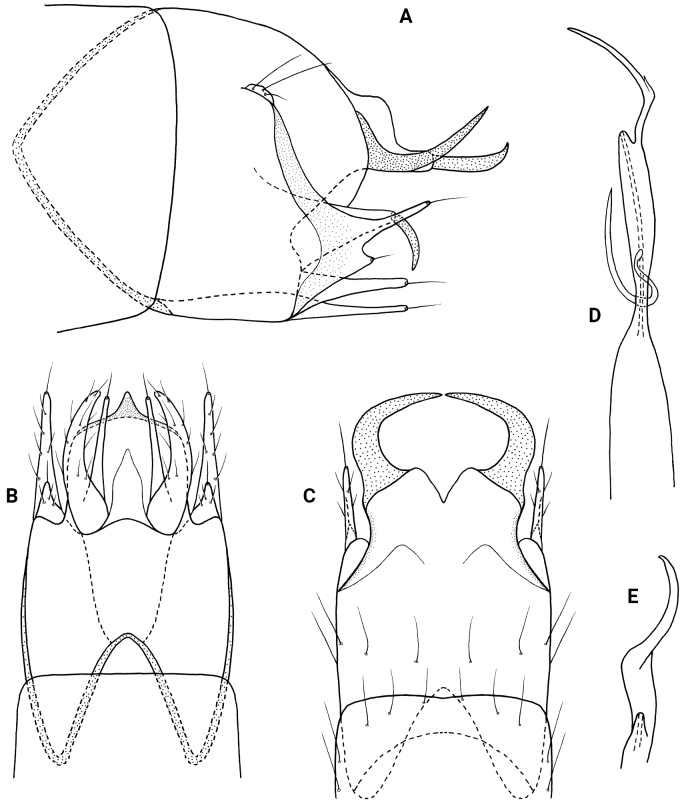
*Neotrichiamindyae* sp. nov., male holotype, genitalia **A** left lateral **B** ventral **C** dorsal **D** phallus, left lateral **E** phallus apex, dorsal.

**Male.** Total length 1.5 mm (*n* = 1), 18 antennal segments, wings and body brown in alcohol. ***Genitalia*** (Fig. 10). Abdominal segment VIII annular. Segment IX in lateral view generally ovate, rounded anteriorly and posteriorly, fused with segment X dorsally, and bearing lateral setal lobes posteriorly; in ventral view anterior margin deeply incised, posterior margin with triangular mesal extension; in dorsal view with pair of posterolateral lobes. Tergum X basally fused with segment IX, posteriorly produced into pair of thick, symmetrical horns which nearly touch mesally; in lateral view distal horns are thin and tapering to acute apices. Subgenital plate in lateral view, wide basally, tapering distally to downturned apical hook; in ventral view wide basally, rounded laterally to apicomesal extension. Bracteole in lateral view wide anteriorly, bifid posteriorly, dorsal branch elongate with apical seta, ventral branch greatly reduced with apical seta; in ventral and dorsal views both branches wide basally, tapering to rounded apices. Inferior appendage bifid laterally, each thin arm wide basally and tapering distally to rounded apices bearing long seta; in ventral view bifid, outer process wide basally, curving and tapering to acute apices, inner process fused basally and narrow over length which is equal to outer process bearing elongate seta apically. Phallus tubular, wide basally, constricted at midlength and bearing thin paramere encircling shaft, apex with elongate lateral spike, smaller spike near base, ejaculatory duct not protruding.

##### Distribution.

Panama: Darién Province (Darién National Park).

##### Etymology.

This species is named for the sister of the first author, Melinda “Mindy” Harris Haupt, who used to help her brother collect bugs when they were both much younger. The name is a noun in the genitive case.

#### ﻿ Neotrichia panamensis sp. nov.

Taxon classificationAnimaliaTrichopteraHydroptilidae

EB24B2D0-24AD-5C9E-A538-CDC18711FE3A

https://zoobank.org/11AF342D-DBD6-46E8-B186-C7F90A9A4E48

Fig. 11

##### Type locality.

**Panama: Colon Province**: Cuenca 117, Portobelo District, Quebrada sin nombre, nr Jose Pobre property–Tesoro Verde; 9.60069°N, 79.61658°W; 55 m a.s.l.

##### Type material.

***Holotype***: ♂, **Panama: Colon Province**: Cuenca 117, Portobelo District, Quebrada sin nombre, nr Jose Pobre property–Tesoro Verde; 9.60069°N, 79.61658°W; 55 m a.s.l.; 19.xii.2018; D. Garrido leg.; UV light trap; MUPADI-012-T-2023 (in alcohol). ***Paratypes*: Panama** • 2 ♂♂; same as holotype; MUPADI-013-T-2023 (in alcohol).

##### Other material examined.

**Panama** • 16 ♂♂; **Veraguas Province**: Cuenca 116, Las Palmas District, Quebrada La Mina; 7.87443°N, 81.51004°W; 63 m a.s.l.; 3.ii.2023; V. Rodríguez leg.; UV light trap • 7 ♂♂; ibid., except Río Indio; 7.87372°N, 81.49994°W; 57 m a.s.l.; 3.ii.2023 • 1 ♂; ibid., except Río Pixvae; 7.84287°N, 81.56329°W; 17 m a.s.l.; 23.i.2023 • 4 ♂♂; ibid., except Soná District, Quebrada Monita; 7.81480°N, 81.55724°W; 26 m a.s.l.; 21.i.2023.

##### Diagnosis.

*Neotrichiapanamensis* sp. nov. is another member of the *N.canixa* group of Keth et al. (2015) based on the posterior horns from tergum X, forked bracteole, and the bifid inferior appendage. The new species appears to be similar to *N.alsa* Oláh & Johanson, 2011 from Peru on the basis of the bracteoles and phallic apex, but the short inferior appendages are more like those of *N.tauricornis* Malicky, 1980 which occurs throughout the Caribbean islands, Panama, and Colombia, but the subgenital plate and bracteoles are much different in the new species compared to that of *N.tauricornis*.

##### Description.

**Figure 11.**
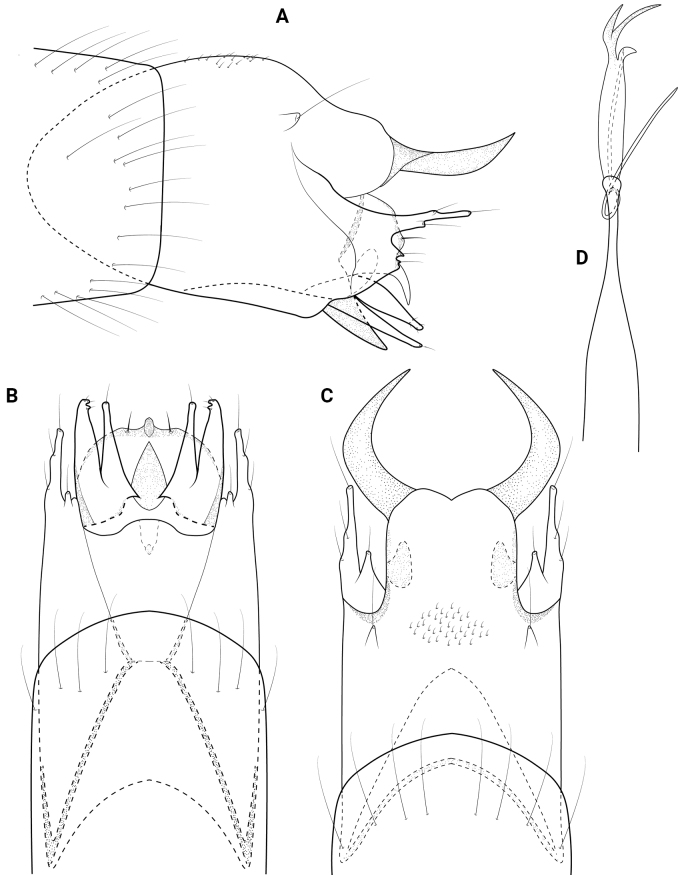
*Neotrichiapanamensis* sp. nov., male holotype, genitalia **A** left lateral **B** ventral **C** dorsal **D** phallus, dorsal.

**Male.** Total length 1.5–1.7 mm (*n* = 14), 18 antennal segments, wings and body brown in alcohol. ***Genitalia*** (Fig. 11). Abdominal segment VIII annular. Segment IX in lateral view ovate, rounded anteriorly and sinuate posteriorly, fused with segment X dorsally, bearing a setae-bearing lobe dorsally; in ventral view anterior margin deeply incised, posterior margin sinuate, triangular mesal extension. Tergum X basally fused with segment IX, rectangular basally, pair of thin, symmetrical horns distally; in lateral view segment X is lobate, with distal horn saber-like. Subgenital plate in lateral view, wide basally, truncate distally with ventral hook tapering to acute apex; in ventral view wide basally, slightly curving to rounded apex, with mesal process flanked by stout setae. Bracteole in lateral view wide anteriorly, slightly bifid posteriorly, dorsal branch elongate and tipped with seta, ventral branch vestigial, represented by pair of sharp points; in ventral and dorsal views lower branch represented by short knob, tapering distally. Inferior appendage short and bifid, wide basally tapering distally, basal process triangular; in ventral view bifid, outer process subrectangular, apex with mesal points, inner process same length as outer, fused and wide at base, tapering distally bearing elongate seta apically. Phallus tubular, constricted at midlength and bearing thin paramere encircling shaft, apex divided into three processes, lower process small, distally processes elongate, ejaculatory duct not protruding at base.

##### Distribution.

Panama: Colon Province (Portobelo District); Veraguas Province (Las Palmas District).

##### Etymology.

This species is named for the Republic of Panama where the species was collected. The name is a noun in the genitive case.

#### ﻿ Neotrichia parajarochita sp. nov.

Taxon classificationAnimaliaTrichopteraHydroptilidae

35B3BDD4-4956-58A8-A6CB-72C0F4202F77

https://zoobank.org/5015E444-2B3B-45EF-A0AF-0506BCA35FA9

Fig. 12

##### Type locality.

**Panama: Chiriquí Province**: Cuenca 102, Bugaba District, afluente Río Chiriquí Viejo, PILA; PSPSCB-PILA-C102-2017-022; 8.90124°N, 82.61817°W; 2354 m a.s.l.

##### Type material.

***Holotype***: ♂, **Panama: Chiriquí Province**: Cuenca 102, Bugaba District, afluente Río Chiriquí Viejo, PILA; PSPSCB-PILA-C102-2017-022; 8.90124°N, 82.61817°W; 2354 m a.s.l.; 17–21.vi.2017; E. Álvarez, E. Pérez, T. Ríos leg.; Malaise trap; MIUP-019-T-2023 (in alcohol).

##### Diagnosis.

*Neotrichiaparajarochita* sp. nov. is another member of the *N.canixa* group of Keth et al. (2015) based on the posterior horns from tergum X, forked bracteole, and the forked apex of the phallus. In many respects, the new species appears to be similar to *N.jarochita* Bueno-Soria, 1999 and *N.palitla* Harris & Flint, 2016, both of which occur in Mexico, on the basis of the asymmetrical horns of tergum X and the structure of the bracteole. Unlike these two species, *N.parajarochita* has a sclerotized posterolateral process from segment IX and the inferior appendages are divergent in ventral view.

##### Description.

**Figure 12.**
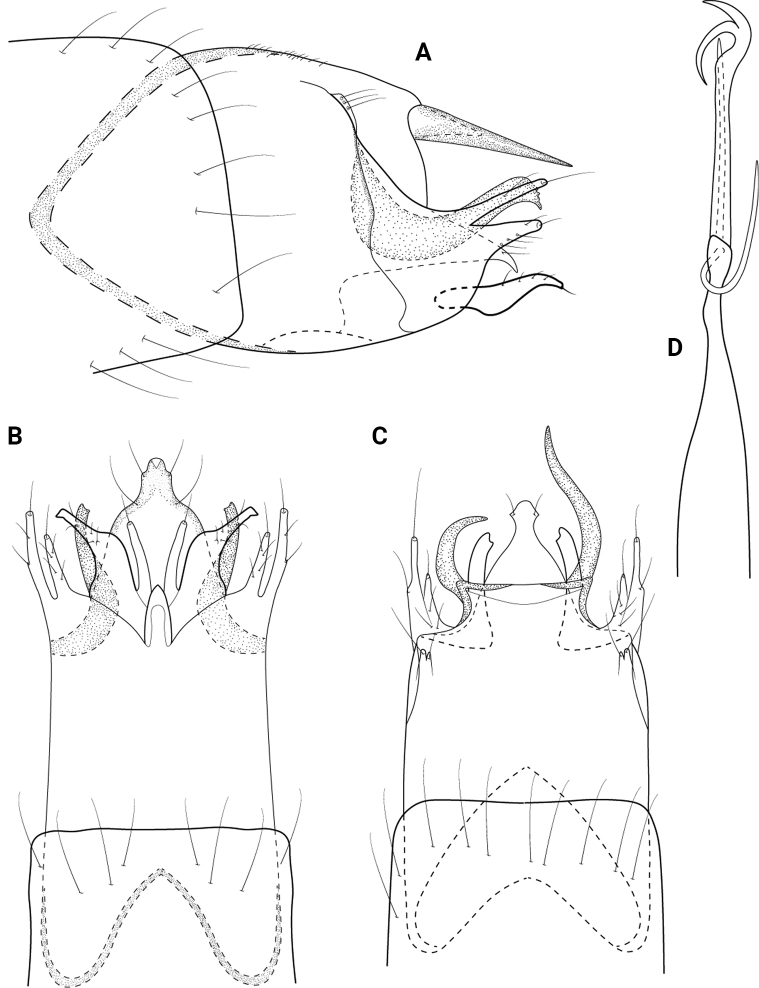
*Neotrichiaparajarochita* sp. nov., male holotype, genitalia **A** left lateral **B** ventral **C** dorsal **D** phallus, ventral.

**Male.** Total length 1.4 mm (*n* = 1), 18 antennal segments, wings and body brown in alcohol. ***Genitalia*** (Fig. 12). Abdominal segment VIII annular. Segment IX in lateral view ovate, rounded anteriorly and sinuate posteriorly, setae-bearing lobe dorsally and fused with segment X, with upturned posterolateral process mesally, narrowing distally to serrate apex; in ventral view anterior margin emarginate, posterior margin incised mesally, with an oval mesal extension, and posterolateral processes laterad, narrow over length and curving. Tergum X basally fused with segment IX, quadrate, posterior horns asymmetrical, left horn twice as long as right; in lateral view segment X is truncated, distal horn elongate and tapering to acute apex. Subgenital plate in lateral view, wide basally, tapering distally to acute apex, which is slightly downturned; in ventral view roundly triangular, abruptly narrowed distally forming rectangular mesal extension flanked by pair of setae. Bracteole in lateral view wide anteriorly, bifid posteriorly, thin dorsal branch slightly longer than triangular ventral branch, each tipped with seta; in ventral and dorsal views wide basally, tapering distally. Inferior appendage short in lateral view, widening at midlength, then tapering to rounded apex; in ventral view bifid, outer process wide basally, narrowing distally and strongly diverging, rectangular apically with lateral spike, inner process shorter and narrower than outer process. Phallus tubular, constricted at midlength and bearing thin paramere encircling shaft, apex forked, lower fork strongly curving, ejaculatory duct protruding subapically.

##### Distribution.

Panama: Chiriquí Province (Parque International La Amistad).

##### Etymology.

This species is named for its resemblance to *Neotrichiajarochita*. The name is an adjective used as a substantive in the genitive case.

#### ﻿ Neotrichia paraxicana sp. nov.

Taxon classificationAnimaliaTrichopteraHydroptilidae

7C9F1E29-6801-5FA0-8CEA-174746CEE289

https://zoobank.org/B577FA98-7BCD-4901-AE56-655F8843BE52

Fig. 13

##### Type locality.

**Panama: Veraguas Province**: Cuenca 132, Santa Fe District, Santa Fe NP, Quebrada Mulabá, Santa Fe NP, PSPSCB-PNSF-C132-2017-009; 8.52560°N, 81.12956°W; 623 m a.s.l.

##### Type material.

***Holotype***: ♂, **Panama: Veraguas Province**: Cuenca 132, Santa Fe District, Santa Fe NP, Quebrada Mulabá, Santa Fe NP, PSPSCB-PNSF-C132-2017-009; 8.52560°N, 81.12956°W; 623 m a.s.l.; 20.iv.2017; A. Cornejo, T. Ríos, C. Nieto leg.; UV light trap; MIUP-020-T-2023 (in alcohol).

##### Other material examined.

**Panama** • ♂; **Veraguas Province**: Cuenca 097, Santa Fe District, Santa Fe NP, Río Piedra de Moler; PSPSCB-PNSF-C097-2017-012; 8.56553°N, 81.18817°W; 340 m a.s.l.; 20.iv.2017; A. Cornejo, T. Ríos, E. Álvarez, C. Nieto leg.; UV light trap • ♂; **Panama Province**: Panama Canal, date and locality of collection illegible on label; D. Denning leg.

##### Diagnosis.

*Neotrichiaparaxicana* sp. nov. is another member of the *N.canixa* group of Keth et al. (2015) based on the posterior horns from tergum X, forked bracteole, bifid inferior appendage, and the forked phallic apex. The new species is most similar to *N.xicana* (Mosely, 1937), reported from Mexico and Panama (Holzenthal and Calor 2017), based on both having a posterolateral process from segment IX, which is lobate apically. However, in the new species this process is very short, and the phallic apex is multi-forked.

##### Description.

**Figure 13.**
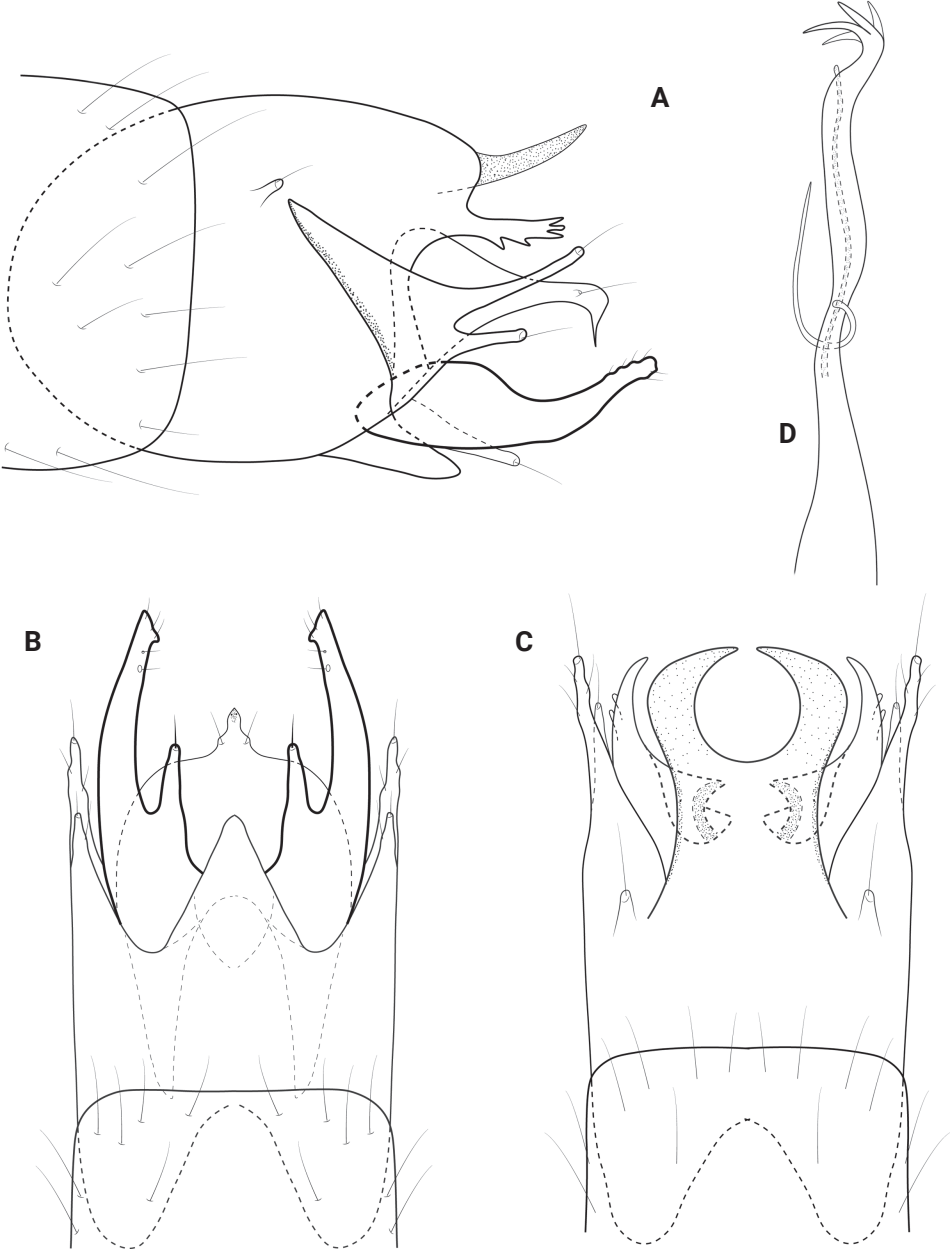
*Neotrichiaparaxicana* sp. nov., male holotype, genitalia **A** left lateral **B** ventral **C** dorsal **D** phallus, left lateral.

**Male.** Total length 1.6–1.8 mm (*n* = 3), 18 antennal segments, wings and body brown in alcohol. ***Genitalia*** (Fig. 13). Abdominal segment VIII annular. Segment IX in lateral view generally ovate, rounded anteriorly, setae-bearing lobe dorsally, posteriorly fused with segment X dorsally, incised posteroventrally, short posterolateral process with multiple small lobes apically; in ventral view posterior margin deeply incised laterally, anteriorly margin broadly incised; in dorsal view posterolateral process from IX visible as a curving, tapering process with subapical lobes laterally. Tergum X basally fused with segment IX, posteriorly produced into pair of thick, symmetrical horns; in lateral view distal horn is thin and tapering to acute apex. Subgenital plate in lateral view, wide basally, tapering apically to rounded apex, with acute downward pointing spike; in ventral view wide basally, rounded laterally to apicomesal extension bearing stout setae laterad. Bracteole in lateral view wide anteriorly, bifid posteriorly, dorsal branch ~ 2× as long as ventral branch; in ventral and dorsal views both branches wide basally, tapering to rounded apices. Inferior appendage wide basally with narrow basal process ventrally, abruptly narrowing at midlength and curving upward to thin, truncated apex; in ventral view bifid, outer process elongate, wide basally and tapering to acute apices with inner spike, inner process fused basally and narrow over length, shorter than outer process. Phallus tubular, constricted at midlength and bearing thin paramere encircling shaft, apex divided into four spine-like processes, ejaculatory duct protruding subapically.

##### Distribution.

Panama: Veraguas Province (Santa Fe National Park).

##### Etymology.

This species is named for its overall resemblance to *Neotrichiaxicana*. The name is an adjective used as a substantive in the genitive case.

#### ﻿ Neotrichia snixae sp. nov.

Taxon classificationAnimaliaTrichopteraHydroptilidae

ADA8DC8D-22DB-5E66-A961-274B4B819747

https://zoobank.org/073C61D3-B348-430C-B442-3792E6BBCFFE

Fig. 14

##### Type locality.

**Panama: Chiriquí Province**: Cuenca 102, Renacimiento District, Reserva Privada Landis, Quebrada sin nombre, Location 1; 8.64379°N, 82.82949°W; 755 m a.s.l.

##### Type material.

***Holotype***: ♂, **Panama: Chiriquí Province**: Cuenca 102, Renacimiento District, Reserva Privada Landis, Quebrada sin nombre, Location 1; 8.64379°N, 82.82949°W; 755 m a.s.l.; 15–31.iii.2020; M. Landis leg.; Malaise trap; MUPADI-014-T-2023 (in alcohol).

##### Other material examined.

**Panama** • ♂; **Chiriquí Province**: Cuenca 108, Boquete District, Quebrada Jaramillo Abajo; 8.745827°N, 82.418083°W; 1054 m a.s.l.; 6.ii.2019; K. Castillo leg.; UV light trap.

##### Diagnosis.

*Neotrichiasnixae* sp. nov. is a member of the *N.canixa* group of Keth et al. (2015) based on the posterior horns from tergum X and the forked bracteole. The horns are asymmetrical, which is similar to that seen in *N.malickyi* Harris & Tiemann, 1993 from Panama and *N.jarochita* Bueno-Soria, 1999 from Mexico, both of which have the right horn longer than that of the left. The new species differs in the presence of a sclerotized posterolateral process from segment IX, which is also seen in *N.juani* Harris & Tiemann, 1993 from Texas and *N.unamas* Botosaneanu (in Botosaneanu and Alkins-Koo 1993), which occurs in Panama, and in the shape of the end of the phallus.

##### Description.

**Figure 14.**
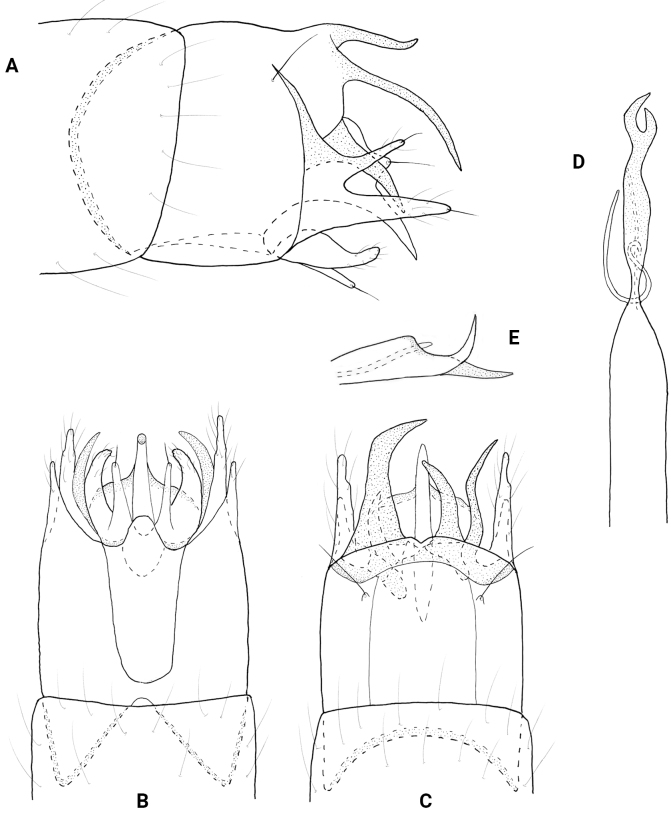
*Neotrichiasnixae* sp. nov., male holotype, genitalia **A**, left lateral **B** ventral **C** dorsal **D** phallus, dorsal **E** phallus apex, lateral.

**Male.** Total length 1.9 mm (*n* = 2), 18 antennal segments, wings and body brown in alcohol. ***Genitalia*** (Fig. 14). Abdominal segment VIII annular. Segment IX in lateral view anteriorly rounded, incomplete posteriorly, fused with X dorsally, mesally with sclerotized posterolateral process which narrows distally; in dorsal view fused posteriorly with X, pair of setaceous lobes laterally, anteriorly with rounded incision, lateral processes dissimilar in length and shape; in ventral view with deep mesal incision. Tergum X basally fused with segment IX, quadrate, posterior horns asymmetrical, right horn longer than left and thicker, curving inward distally; in lateral view ventral horn twice as long as dorsal horn. Subgenital plate in lateral view wide basally, tapering distally to divided apex, dorsally with stout seta, ventrally with elongate downturned process which is apically acute; in dorsal and ventral views wide basally, elongate posteromesally, rounded laterally. Bracteole in lateral view bifid posteriorly, ventral branch longer than dorsal, each tipped with stout seta; in dorsal and ventral views branches unequal in length, wide basally tapering distally. Inferior appendage wide basally, gradually tapering distally to rounded slightly upturned apex, narrow ventral process basally; in ventral view wide basally, curving on inner margin to rounded apex, mesal process tapering distally and bearing stout seta. Phallus tubular, constricted at midlength and bearing thin paramere encircling shaft, apically sclerotized with narrow apical incision forming unequal branches, similar in appearance to a can-opener; in lateral view apex divided into pair of elongate, thin processes.

##### Distribution.

Panama: Chiriquí Province (Reserva Privada Landis; Boquete District).

##### Etymology.

Named for Dr. Shannon Nix, friend and colleague of the first author in recognition of her outstanding teaching while at Clarion University and her many scientific contributions to ecology and mycology. The name is a noun in the genitive case.

#### ﻿ Neotrichia spangleri sp. nov.

Taxon classificationAnimaliaTrichopteraHydroptilidae

2BC41B52-A388-579E-B4BF-17EFD0977AD5

https://zoobank.org/F5ABEE1A-A42C-4192-9A98-1615AD3B938D

Fig. 15

##### Type locality.

**Panama: Chiriquí Province**: Cuenca 108, Boquete District, Bajo Boquete, Quebrada Cheche, Hotel Fundadores; 8.77195°N, 82.43308°W; 1200 m a.s.l.

##### Type material.

***Holotype***: ♂, **Panama: Chiriquí Province**: Cuenca 108, Boquete District, Bajo Boquete, Quebrada Cheche, Hotel Fundadores; 8.77195°N, 82.43308°W; 1200 m a.s.l.; 29.v.1983; P. Spangler, R. Faitoule, W. Steiner leg.; MUPADI-015-T-2023 (in alcohol). ***Paratype*. Panama** • ♂; same as holotype; MUPADI-016-T-2023 (in alcohol).

##### Other material examined.

**Panama** • 2 ♂♂; **Chiriquí Province**: Cuenca 104, Bugaba District, La Concepción, Río Guigala, Puente antiqua vias del Ferrocarril; 8.51845°N, 82.64280°W; 209 m a.s.l.; 12.iii.2021; T. Ríos, Y. Aguirre leg.; UV light trap • 2 ♂♂; ibid., except Cuenca 108, Boquerón District, Río Chirigagua, Puente antes de llegar al Hotel Los Delfines; 8.48139°N, 82.54788°W; 128 m a.s.l.; 12.iv.2021; T. Ríos, Y. Aguirre leg.; UV light trap • 4 ♂♂; ibid., except David District, San Pablo Viejo, puente vía Interamericana antes de llegar a la entrada de Bagala, Río Platanal; 8.46416°N, 82.52030°W; 84 m a.s.l.; 12.ii.2021; T. Ríos, Y. Aguirre leg.; UV light trap • ♂; ibid., except 12.iii.2021 • 2 ♂♂; ibid., except 12.iv.2021 • 11 ♂♂; ibid., except 6.x.2021 • 4 ♂♂; ibid., except 6.xi.2021 • 2 ♂♂; **Darién Province**; Cuenca 156, Chepigana District, PND, Río Tuira, Boca de Cupe; 8.01732°N, 77.72417°W; 150 m a.s.l.; 18.ii.1985; leg. not given; UV light trap (NMNH).

##### Diagnosis.

The pair of spines at the phallic apex and the posterolateral process from segment IX places this species in the *N.collata* group of Keth et al. (2015) with similarity to *N.hiaspa* (Mosely, 1937) and *N.carlsoni* Harris & Armitage, 2019, both of which occur in Panama. The new species is separated by the shorter and wider inferior appendage, the rounded posterolateral process from segment IX, which is thin and acute in the other species, and the structure of the subgenital plate.

##### Description.

**Figure 15.**
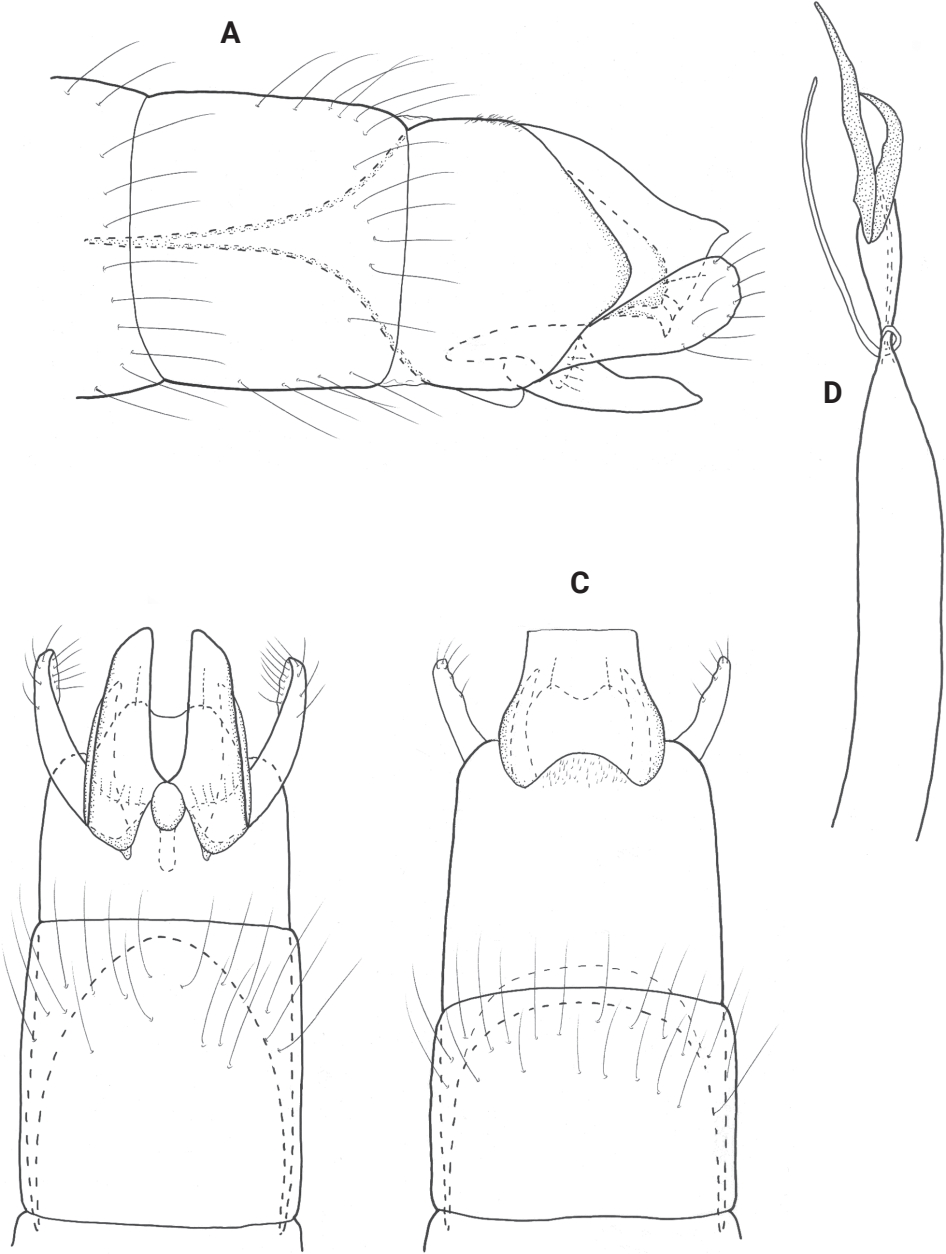
*Neotrichiaspangleri* sp. nov., male holotype, genitalia **A** left lateral **B** ventral **C** dorsal **D** phallus, dorsal.

**Male.** Total length 1.3–1.5 mm (*n* = 10), 18 antennal segments, wings and body brown in alcohol. ***Genitalia*** (Fig. 15). Abdominal segment VIII annular. Segment IX in lateral view anteriorly tapering to an elongate apodeme, posteriorly widening mesally, which gives rise to a rounded posterolateral process; ventrally deeply incised anteriorly, posteriorly with lateral incisions forming mesal ovate structure; posterolateral processes thin, tapering to acute apices; dorsally with posterior shallowly incised laterally, setose mesally. Segment X in lateral view tapering to acute distal point; in dorsal view wide basally, rounded laterally to truncate apex. Subgenital plate in lateral view narrow, apex downturned and acute; in dorsal and ventral view wide basally, apex with mesal emargination, flanked by pair of setae. Bracteole in lateral view narrow basally, widening distally to rounded apex; in ventral and dorsal views nearly parallel-sided, curving on outer margin. Inferior appendage short, wide basally, tapering distally to broadly pointed apex; in ventral view rectangular, apex tapered to rounded point. Phallus in dorsal view tubular, bearing short paramere encircling shaft at midlength, apex with pair of elongate sclerotized rods fused basally, one rod ~ 1/2 length of other and curved.

##### Distribution.

Panama: Chiriquí Province (Boquerón, Boquete, and Bugaba districts); Darién Province (Chepigana District).

##### Etymology.

The species name honors the memory of Dr Paul Spangler of the National Museum of Natural History, who collected some of the specimens. The name is a noun in the genitive case.

#### ﻿ Neotrichia veraguasensis sp. nov.

Taxon classificationAnimaliaTrichopteraHydroptilidae

0A3E44B9-223D-5FBF-A51A-D12CC7634A26

https://zoobank.org/215B4D9D-9270-49E4-8481-3F273FC34106

Fig. 16

##### Type locality.

**Panama: Veraguas Province**: Cuenca 132, Santa Fe District, Santa Fe NP, Quebrada Mulabá; PSPSCB-PNSF-C132-2017-009; 8.52560°N, 81.12956°W; 623 m a.s.l.

##### Type material.

***Holotype***: ♂, **Panama: Veraguas Province**: Cuenca 132, Santa Fe District, Santa Fe NP, Quebrada Mulabá; PSPSCB-PNSF-C132-2017-009; 8.52560°N, 81.12956°W; 623 m a.s.l.; 20.iv.2017; A. Cornejo, T. Ríos, E. Álvarez, C. Nieto leg.; UV light trap; MIUP-021-T-2023 (in alcohol).

##### Other material examined.

**Panama** • ♂; ibid., except Río Piedra de Moler; PSPSCB-PNSF-C097-2017-012; 8.56553°N, 81.18817°W; 340 m a.s.l.; 20.iv.2017; MIUP (in alcohol).

##### Diagnosis.

This new species, which lacks spines at the phallic apex, is placed in the *N.okopa* group of Keth et al. (2015), with closest similarity to *N.abbreviata* Flint, 1983 from Uruguay and *N.okopa* Ross, 1939 from throughout North America. The new species is separated by the triangular inferior appendage in lateral view, and the lack of a posterolateral process from segment IX.

##### Description.

**Figure 16.**
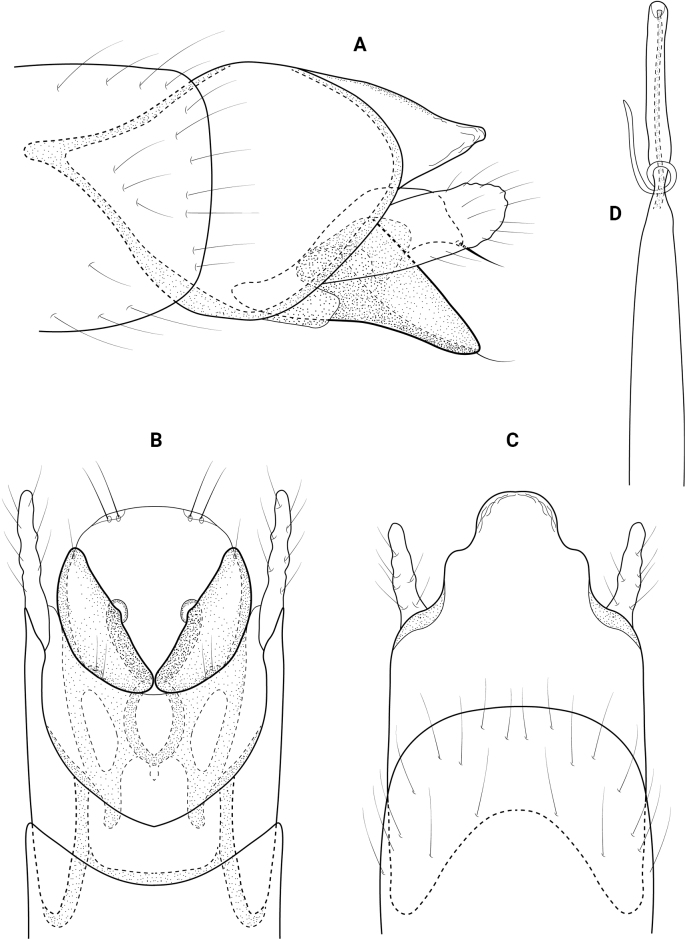
*Neotrichiaveraguasensis* sp. nov., male holotype, genitalia **A** left lateral **B** ventral **C** dorsal **D** phallus, ventral.

**Male.** Total length 1.6 mm (*n* = 2), 18 antennal segments, wings and body brown in alcohol. ***Genitalia*** (Fig. 16). Abdominal segment VIII annular. Segment IX in lateral view anteriorly tapering to short apodeme, posteriorly rounded, short truncate lobe posteroventrally; ventrally divided into two posterior bands; dorsally with anterior margin incised, posterior shallowly emarginate. Segment X triangular in lateral view; in dorsal view fused basally with segment IX, posteriorly sinuate on lateral margin and tapering to rounded apex. Subgenital plate in lateral view narrow basally, ventral margin with large sclerotized lobe at midlength, upper margin straight distally, abruptly tapered posteriorly to rounded apex bearing stout seta; in ventral view wide and rounded laterally to truncate apex, with setae laterally. Bracteole in lateral view parallel-sided to rounded apex; in ventral and dorsal views straight, narrow over length. Inferior appendage triangular and sclerotized; in ventral view divergent, lateral margin curving, inner margin straight, round lobe at midlength, basally with elaborate thin sclerites. Phallus in dorsal view tubular, bearing short paramere encircling shaft at midlength, apex rectangular with ejaculatory duct protruding mesally.

##### Distribution.

Panama: Veraguas Province (Santa Fe National Park).

##### Etymology.

This species is named for Veraguas Province where the male holotype was collected. The name is a noun in the genitive case.

### ﻿New country records

#### ﻿ Neotrichia minutisimella

Taxon classificationAnimaliaTrichopteraHydroptilidae

(Chambers, 1873)

4D85C6AB-EF53-53D4-8097-0F53DFDE668E

##### Material examined.

**Panama** • ♂; **Veraguas Province**: Cuenca 097, PNSF, afluente sin nombre de Río Calovebora; PSPSCD-PNSF-CO97-2017-011; 8.55343°N, 81.17675°W; 395 m a.s.l.; 20.iv.2017; A. Cornejo, T. Ríos, E. Álvarez, C. Nieto leg.; UV light trap.

##### Note.

This is a new country record for Panama and a significant extension of its southern range (formerly Texas, U.S.A.).

##### Distribution.

Canada: Manitoba; Panama: Veraguas Province (Santa Fe National Park); U.S.A.: Alabama, Arkansas, Florida, Georgia, Illinois, Indiana, Kansas, Kentucky, Louisiana, Minnesota, Mississippi, Missouri, North Carolina, Oklahoma, South Carolina, Texas.

#### ﻿ Neotrichia vibrans

Taxon classificationAnimaliaTrichopteraHydroptilidae

Ross, 1944

6B49EE01-F3F6-5F43-BF0E-D8F3F91A6DDB

Figs 17 , 18

##### Material examined.

**Panama** • 27 ♂♂, 25 ♀♀; **Chiriquí Province**: Cuenca 108, David, UNACHI–Jardin Botanico, El Cabrero, nr Quebrada San Cristobal; 8.434060°N, 82.451930°W; 45 m a.s.l.; 19.iv–3.v.2021; Y. Aguirre, T. Ríos leg.; Malaise trap • 22 ♂♂, 23 ♀♀; ibid., except 5–19.iv.2021 • ♂; **Panama Oeste Province**: Cuenca 115, Chame District, Altos de Campana NP, Río Sajalices; PSPSCB-PNAC-C115-2018-030; 8.67625°N, 79.89748°W; 194 m a.s.l.; 27–31.v.2018; E. Pérez, C. Nieto, M. Molinar, T. Ríos leg.; Malaise trap.

**Female** (Fig. 18). The female of *N.vibrans* is slightly larger than the males, with total length from 2.0 to 2.5 mm compared to 1.8 to 2.0 mm, both having 18 antennal segments and the wings and body are brown in alcohol. Abdominal sternite VIII has a heavily sclerotized square plate (Fig. 18A), with a thin ridge on the posterior margin that narrows to an acute posteromesal point, which projects downward in lateral view, anteriorly the plate margin is rounded with a thin sinuate internal structure. The bursa copulatrix lies under the sclerotized plate of segment VIII and details are difficult to discern, but it is generally rectangular in shape, the genital chamber narrowing posteriorly to an acute point, genital chamber broadly incised anteriorly producing a pair of “feet” with heavily sclerotized inner margins, these “feet” extend posteriorly forming a mesal sclerite (Fig. 18B).

##### Note.

**Figure 17.**
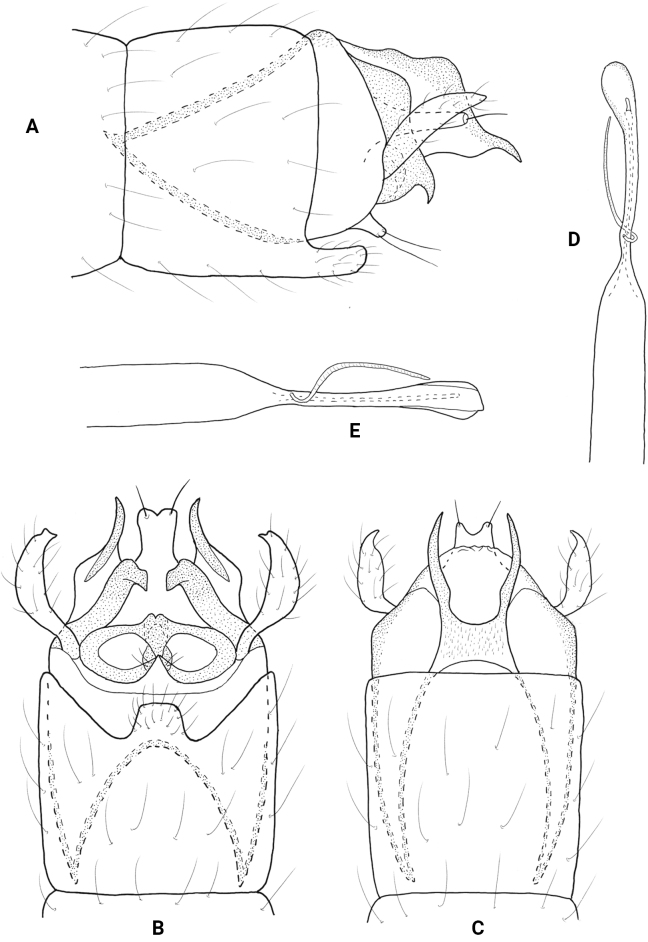
*Neotrichiavibrans* Ross, male genitalia **A** left lateral **B** ventral **C** dorsal **D** phallus, dorsal **E** phallus, lateral.

This is a new country record for Panama. In addition, it is a significant southern extension of the species range, which formerly was northern Mexico. The male of this species is here re-illustrated (Fig. 17) because the figures in Keth et al. (2015) do not clearly depict the lateral view of this species. The posterolateral processes from abdominal segments IX and X are difficult to see in those drawings and the bracteole is obscured by the process from IX. As well, the figure for *N.vibrans* in Ross (1944) lacks a lateral view and the phallus is shown as the apex having a pair of lobes bearing stout spines. These lobes and spines, which are also used as characters in the diagnostic key in Ross (1944), belong to the apex of the subgenital plate, with the phallus lacking apical spines.

**Figure 18.**
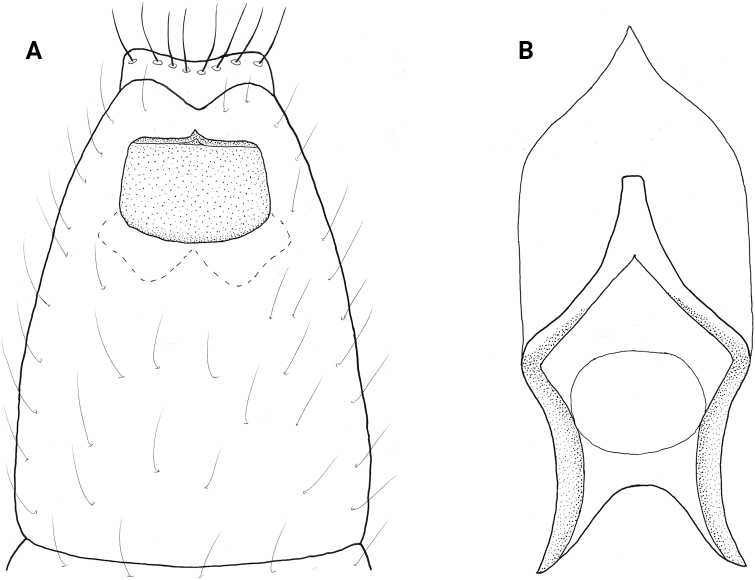
*Neotrichiavibrans* Ross, female genitalia **A** ventral **B** bursa copulatrix, ventral.

Females of other species from the *N.vibrans* group of Keth et al. (2015) also have a sclerotized plate on the sternum of segment VIII, including *Neotrichia* ♀ sp. B (in Botosaneanu and Alkins-Koo 1993), which was tentatively identified as *N.armata* Botosaneanu, 1993, *N.iridescens* Flint, 1964, and *N.soleaferrea* Botosaneanu (in Botosaneanu and Hyslop 1998). However, other members of this group have the females unidentified, or in the case of *N.heleios* Flint, 1968 the sternum of sternite VIII lacks a sclerotized plate. Also, with the exception of *N.soleaferrea*, the structure of the bursa copulatrix appears to be much different than that seen in *N.vibrans*.

The ventral plate on sternite VIII is not a characteristic unique to the *N.vibrans* group. Females in other groups have a distinctive sclerotized plate, e.g., *N.margararitena* Botosaneanu (in Botosaneanu and Viloria 2002) of the *N.biuncifera* group (Marshall 1979). Also, females for several members of the *N.caxima* group, including *N.nesiotes* Flint & Sykora, 1993, *N.mentonensis* Frazer & Harris, 1991, *N.rasmusseni* Harris & Keth, 2002, and *N.armitagei* Harris, 1991 share this trait. In addition, many members of the *N.caxima* group also have females with the structure of the bursa copulatrix very similar to that seen in the female of *N.vibrans* (Harris and Rasmussen 2010).

Considering the above variation and overlap of characters, more associations are needed to facilitate a detailed comparison and delineation of *Neotrichia* females before we can adequately diagnose any females definitively. The descriptive text for *N.vibrans* above is a contribution toward that future comparison. Finally, we were fortunate in being able to associate the female of *N.vibrans* with the male because individuals of both sexes were found, in quantity, together in the absence of any other congeners in a non-natural botanical garden site. Most natural stream sites we have sampled in Panama have 2–7 species present in the same sample, making associations more difficult.

##### Distribution.

Mexico: Chihuahua; Panama: Chiriquí Province (David District); U.S.A.: Alabama, Arkansas, Florida, Georgia, Illinois, Kansas, Kentucky, Maine, Minnesota, Mississippi, Missouri, New Hampshire, New York, Ohio, Oklahoma, South Carolina, Tennessee, Texas, Virginia, West Virginia, Wisconsin.

### ﻿Key to the males of *Neotrichia* in Panama

Species of *Neotrichia* are difficult to identify given their tiny size, which varies from 1 to 3 mm, and the complex genitalia, on which identifications are dependent. The small size often necessitates that specimens are mounted on slides and observed at high magnification. Drawings are likewise prepared from these slide-mounted specimens. Two broad characters are often useful in making species determinations: small terminal horns located on the apex of tergum X and the presence of a posterolateral process from segment IX, which is often sclerotized. When the terminal horns are present, characters of the bracteoles, inferior appendages and phallus become important. When a posterolateral process from segment IX is present, then the appearance of these processes and their location and point of origin, the bracteoles, phallus, and inferior appendages all provide useful characters. For example, the posterolateral process can be mesal as in *N.honda* and *N.parajarochita*, dorsal as in *N.landisae* and *N.paraxicana*, and ventral as in *N.lenati*. In some instances, what appears to be a process from segment IX is actually a part of the subgenital plate, or another structure. For simplicity and ease of using this key, we have termed what appears to be a posterior extension of segment IX as a posterolateral process.

**Table:** 

1	Inferior appendages fused mesally into a plate, which is evident in ventral view (Fig. 17B; Keth et al. 2015: fig. 85C)	**2**
–	Inferior appendages separate, not fused mesally as seen in ventral view (Figs 2B, 4B, 13B, 15B)	**3**
2	Inferior appendages fused into an elongate rectangular plate (Keth et al. 2015: fig. 85C); abdominal segment VIII without a ventromesal process (Keth et al. 2015: fig. 85A)	***N.minutisimella* (Chambers, 1873)**
–	Inferior appendages fused into an ovate plate (Fig. 17B); abdominal segment VIII with a prominent ventromesal process (Fig. 17A, B)	***N.vibrans* Ross, 1944**
3	Horns from posterior tergum X, which vary in shape and size (Figs 10C, 12C, 14C), and may be symmetrical (Figs 3C, 13C) or asymmetrical (Figs 12C, 14C); phallus sharply narrowing apically, often bifid and recurving (Figs 3D, 11D, 12D, 13D); bracteoles typically bifid (Figs 3A, 10A, 12A, 14A)	**4**
–	Horns absent from posterior tergum X (Figs 2C, 4C, 5C, 6C), although in some species the posterolateral projection from segment IX may appear to be horn-like (Fig. 8A; Armitage and Harris 2018: fig. 7A, B; Harris and Armitage 2019: fig. 16A); phallus apex various, but typically not bifid and recurved (Figs 2D, 4D, 6D, 9D, 15D), bracteoles various, but not typically bifid (Figs 2A, 4A, 5A, 7A, 15A, 16A)	**20**
4	Horns from posterior tergum X asymmetrical (Figs 12C, 14C)	**5**
–	Horns from posterior tergum X symmetrical (Figs 3C, 10C, 11C)	**7**
5	Left-side horn of posterior tergum X longer than right-side horn (Fig. 12C); posterolateral process from segment IX present (Fig. 12A)	***N.parajarochita* sp. nov.**
–	Right-side horn of posterior tergum X longer than left-side horn (Fig. 14C, Harris and Tiemann 1993: fig. 2C); posterolateral process from segment IX present (Fig. 14A) or absent (Harris and Tiemann 1993: fig. 2A	**6**
6	Posterolateral process from segment IX present; ventral branch of bracteole longer than dorsal branch (Fig. 14A)	***N.snixae* sp. nov.**
–	Posterolateral process from segment IX absent (Harris and Tiemann 1993: fig. 2A); dorsal branch of bracteole longer than ventral branch (Harris and Tiemann 1993: fig. 2A)	***N.malickyi* Harris, 1993 (in Harris and Tiemann 1993)**
7	Linear posterior process from segment IX present (Fig. 13A)	**8**
–	Linear posterior process from segment IX absent (Armitage and Harris 2018: figs 5A, 6A)	**12**
8	Linear posterior process from segment IX short, not extending beyond horns in lateral view (Fig. 13A)	***N.paraxicana* sp. nov.**
–	Linear posterior process from segment IX elongate, extending beyond horns in lateral view (Harris and Armitage 2019: fig. 15A; Keth et al. 2015: fig. 22A)	**9**
9	Linear posterior process from segment IX serrate (Harris and Armitage 2019: fig. 15A), or lobate or cleft distally (Keth et al. 2015: fig. 22A)	**10**
–	Linear posterior process from segment IX not serrate, or lobate or cleft distally (Fig. 11A, Keth et al. 2015: fig. 21A)	**11**
10	Linear posterior process from segment IX in lateral view serrate on dorsal margin (Harris and Armitage 2015: fig. 14A); subgenital plate in lateral view gradually tapering distally (Harris and Armitage 2015: fig. 15A); inferior appendage in ventral view with outer process curving inward at apex, inner process elongate (Harris and Armitage 2015: fig. 15B)	***N.serrata* Harris & Armitage, 2019**
–	Linear posterior process from segment IX not serrate on distal margin, cleft or lobate distally (Keth et al. 2015: fig. 22A); subgenital plate in lateral view abruptly curving ventrad apically (Keth et al. 2015: fig. 22A); inferior appendage in ventral view with outer process straight, inner process short (Keth et al. 2015: fig. 22B)	***N.xicana* (Mosely, 1937)**
11	Linear posterior process from segment IX widening distally and setose on margin (Keth et al. 2015: fig. 11A); subgenital plate in lateral view narrowing distally (Keth et al. 2015: fig. 11A); small rounded median process between inferior appendages in ventral view (Keth et al. 2015: fig. 11C)	***N.canixa* (Mosely, 1937)**
–	Linear posterior process from segment IX saber-like, narrowing distally and without setose margin (Keth et al. 2015: fig. 21A); subgenital plate in lateral view widening distally and truncate (Keth et al. 2015: fig. 21A); large triangular process between inferior appendages in ventral view (Keth et al. 2015: fig. 22C)	***N.unamas* Botosaneanu, 1993 (in Botosaneanu and Alkins-Koo 1993)**
12	Dorsal branch of bracteole shorter than ventral branch (Keth et al. 2015: fig. 20A); subgenital plate trifid distally in lateral view, ventral-most branch elongate (Keth et al. 2015: fig. 20A)	***N.tauricornis* Malicky, 1980**
–	Dorsal branch of bracteole longer than ventral branch, which may be vestigial (Figs 3A, 10A, 13A); subgenital plate various, but not trifid distally (Figs 10A, 13A)	**13**
13	Bracteole with ventral branch ≥ ½ as long as dorsal branch (Fig. 3A, Armitage and Harris 2018: fig. 6A)	**14**
–	Bracteole with ventral branch < ½ as long as dorsal branch and in some cases vestigial (Figs 10A, 11A)	**17**
14	Inferior appendage in lateral view with dorsal hump at midlength (Armitage and Harris 2018: fig. 6A); horns of tergum X elongate and deeply divided to base (Armitage and Harris 2018: fig. 6B)	***N.collierorum* Armitage & Harris, 2018**
–	Inferior appendage in lateral view parallel-sided (Armitage and Harris 2018: figs 3A, 5A); horns of tergum X shorter and not deeply divided to base (Armitage and Harris 2018: figs 3C, 5B)	**15**
15	Phallus apex undivided, narrowing to elongate slender process (Armitage and Harris 2018: fig. 5D); ventral branch of bracteole short (Armitage and Harris 2018: fig. 5A)	***N.anzuelo* Armitage & Harris, 2018**
–	Phallus apex divided (Harris et al. 2023: figs 3D, 4D); ventral branch of bracteole elongate (Harris et al. 2023: figs 3A, 4A)	**16**
16	Inferior appendage in lateral view saber-shaped (Harris et al. 2023: fig. 4A), ventrally narrower at base than at midlength (Harris et al. 2023: fig. 4B); phallus apex with serrations on upper branch (Harris et al. 2023: fig. 4D); subgenital plate in lateral view narrowing distally and projecting ventrad (Harris et al. 2023: fig. 4A)	***N.majagua* Harris, Ríos & Aguirre, 2023**
–	Inferior appendage in lateral view cigar-shaped (Fig. 3A), ventrally wider at base than at midlength (Fig. 3B); phallus apex without serrations on upper branch (Fig. 3D); subgenital plate in lateral view can-opener shape (Fig. 3A)	***N.candela* sp. nov.**
17	Phallus apex with single elongate process with small notch near base (Fig. 10D, Armitage and Harris 2020: fig. 10D, E)	**18**
–	Phallus apex clearly divided into two processes (Fig. 11D, Harris and Armitage 2015: fig. 4D)	**19**
18	Posterior horns from tergum X strongly curving inward (Fig. 10C); phallus apex in dorsal view with elongate thin process (Fig. 10D); median process between inferior processes in ventral view large and pointed distally (Fig. 10B)	***N.mindyae* sp. nov.**
–	Posterior horns from tergum X not strongly curving inward (Armitage and Harris 2020: fig. 10B); phallus apex in dorsal view with short curving process (Armitage and Harris 2020: fig. 10E); median process between inferior appendages in ventral view short and rounded distally (Armitage and Harris 2020: fig. 10C)	***N.michaeli* Armitage & Harris, 2020**
19	Dorsal branch of inferior appendage in lateral view curving and widening apically (Harris and Armitage 2015: fig. 4A); inferior appendages in ventral view with outer processes longer than inner processes and curving, median process short and truncate (Harris and Armitage 2015: fig. 4C)	***N.pamelae* Harris & Armitage, 2015**
–	Dorsal branch of inferior appendage in lateral view not curving and widening sub-basally (Fig. 11A); inferior appendages in ventral view with outer process equal in length to inner processes and straight, median process elongate and triangular (Fig. 11B)	***N.panamensis* sp. nov.**
20	Posterolateral process from segment IX, which takes different forms, from elongate (Fig. 8A–C; Harris and Armitage 2019: fig. 16A–C) to short (Figs 5A, 15A) or lobate (Figs 6A, 9A), usually this process can be seen in both lateral and dorsal view, and is typically sclerotized (Figs 7A–C, 8A–C)	**21**
–	Posterolateral process from segment IX absent (Fig. 16A–C; Harris 1990: fig. 8A; Thomson and Armitage 2018: fig. 8A)	**36**
21	Posterolateral process from segment IX elongate, exceeding length of inferior appendage and bracteole, heavily sclerotized and narrowing distally to an acute point (Fig. 8A; Harris and Armitage 2019: fig. 16A; Armitage and Harris 2018: fig. 7A)	**22**
–	Posterolateral process from segment IX shorter, typically not exceeding length of inferior appendage and bracteole (Fig. 15A), although it may be spinose (Harris and Armitage 2019: fig. 12A); if longer than bracteole and inferior appendage then not spinose (Fig. 6A; Harris et al. 2023: fig. 6A)	**26**
22	Inferior appendage in lateral view wide basally, greatly tapering distally to uniform width (fig. 13A in Harris and Armitage 2019); phallus apex divided into elongate spines (Harris and Armitage 2019: fig. 13D, E)	***N.hiaspa* (Mosely, 1937)**
–	Inferior appendage various but not wide basally and tapering distally (Fig. 8A; Armitage and Harris 2018: fig. 7A); phallus apex various, but not divided into elongate spines (Fig. 8D)	**23**
23	Elongate, thin ventroposterior process from segment XI; subgenital plate in lateral view with elongate ventral process (Armitage and Harris 2020: fig. 12A)	***N.yayas* Armitage & Harris, 2020**
–	Elongate, thin, ventroposterior process from segment IX absent (Fig. 8A); subgenital plate in lateral view various, but without elongate ventral process (Fig. 8A)	**24**
24	Posterolateral processes from segment IX nearly symmetrical in dorsal view (Fig. 8B; Harris and Armitage 2019: fig. 15C); inferior appendage in lateral view not deeply incised dorsally at midlength (Fig. 8A; Harris and Armitage 2019: fig. 16A)	**25**
–	Posterolateral processes from segment IX asymmetrical in dorsal view (Armitage and Harris 2018: fig. 7B); inferior appendage in lateral view deeply incised dorsally at midlength (Armitage and Harris 2018: fig. 7A)	***N.tatianae* Armitage & Harris, 2018**
25	Posterolateral process from segment IX sinuate in lateral and dorsal view (Harris and Armitage 2019: fig. 16A–C); inferior appendage in lateral view elongate, tapering distally (Harris and Armitage 2019: fig. 16A); bracteole prominent (Harris and Armitage 2019: fig. 16A)	***N.starki* Harris & Armitage, 2019**
–	Posterolateral process from segment IX linear in lateral and dorsal views (Fig. 8A–C); inferior appendage in lateral view short and truncate distally (Fig. 8A); bracteole not prominent (Fig. 8A)	***N.landisae* sp. nov.**
26	Posterolateral process from segment IX spinose, heavily sclerotized and tapering distally (Fig. 7A; Armitage and Harris 2020: fig. 9A; Armitage and Harris 2020: fig. 11A)	**27**
–	Posterolateral process from segment IX various, but not spinose of heavily sclerotized (Figs 4A, 6A, 9A, 15A)	**32**
27	Phallus apex divided into pair of elongate spines (Fig. 7D; Armitage and Harris 2020: fig. 9D, E)	**28**
–	Phallus apex not divided into pair of elongate spines (Armitage and Harris 2020: fig. 11D; Keth et al. 2015: fig. 55B)	**29**
28	Segment IX in lateral view with elongate mesal process (Armitage and Harris 2020: fig. 11A); inferior appendage bifid in lateral and ventral views (Armitage and Harris 2020: fig. 11A, B)	***N.pierpointorum* Armitage & Harris, 2020**
–	Segment IX in lateral view without elongate mesal process (Keth et al. 2015: fig. 55A); inferior appendage rectangular, but not bifid in lateral and ventral views (Keth et al. 2015: fig. 55A–C)	***N.esmalda* (Mosely, 1937)**
29	Inferior appendage in lateral and ventral views elongate and thin, not heavily sclerotized (Harris and Armitage 2019: fig. 12A, B; Armitage and Harris 2020: fig. 9A, B)	**30**
–	Inferior appendage in lateral and ventral views short and triangular, heavily sclerotized Fig. 7A, B)	***N.honda* sp. nov.**
30	Two posterolateral sclerotized processes from segment IX (Armitage and Harris 2020: fig. 9A–C); apical phallic spines equal in length (Armitage and Harris 2020: fig. 9D, E)	***N.espinosa* Armitage & Harris, 2020**
–	Single posterolateral process from segment IX (Harris and Armitage 2019: fig. 12A–C); apical phallic spines unequal in length (Harris and Armitage 2019: fig. 12D, E)	***N.carlsoni* Harris & Armitage, 2019**
31	Phallus with pair of sclerotized spines (Figs 4D, E, 5D, E, 15D)	**32**
–	Phallus with single sclerotized spine (Fig. 6D; Harris et al. 2023: fig. 15D, E) or lacking sclerotized spines (Fig. 9D)	**34**
32	Phallic apical spines terminal (Figs 5D, E, 15D); inferior appendage shorter than bracteole and linear (Figs 6A, 15A)	**33**
–	Phallic apical spines subterminal (Fig. 4D, E); inferior appendage longer than bracteole and angled dorsad at midlength (Fig. 4A)	***N.codaza* sp. nov.**
33	Phallic apical spines short and nearly equal in length (Fig. 5D, E); inferior appendage bifid in lateral and ventral views (Fig. 5A, B)	***N.embera* sp. nov.**
–	Phallic apical spines elongate and subequal in length (Fig. 15D); inferior appendage not bifid in lateral and ventral views (Fig. 15A, B)	***N.spangleri* sp. nov.**
34	Phallus with sclerotized spine (Fig. 6D; Harris et al. 2023: 5D, E); posterolateral process from segment IX elongate, extending beyond bracteole and dorsal in position (Fig. 6A; Harris et al. 2023: fig. 5A)	**35**
–	Phallus without sclerotized spine (Fig. 9D); posterolateral process from segment IX short, not extending beyond bracteole and ventral in position (Fig. 9A)	***N.lenati* sp. nov.**
35	Posterolateral process from segment IX widening distally and serrate (fig. 5A, B in Harris et al. 2023); phallic spine originating apically (Harris et al. 2023: fig. 5D, E)	***N.solapa* Harris, Ríos & Aguirre, 2023**
–	Posterolateral process from segment IX not widening distally or serrate (Fig. 6A, B); phallic spine originating at midlength (Fig. 6D)	***N.flennikeni* sp. nov.**
36	Inferior appendages in lateral view with serrate dorsal process just past midlength (Harris and Armitage 2015: fig. 5A)	***N.parabullata* Harris & Armitage, 2015**
–	Inferior appendages various but never with a serrate dorsal process just past midlength (Figs 2A, 16A; Harris 1990: fig. 8A)	**37**
37	Inferior appendages short in lateral view, not extending much beyond subgenital plate or bracteoles (Fig. 2A; Thomson and Armitage 2018: fig. 6A; Keth et al. 2015: fig. 42A)	**38**
–	Inferior appendages elongate in lateral view, typically extending well beyond the subgenital plate and bracteoles (Fig. 16A; Harris 1990: fig. 8A)	**41**
38	Bracteole in lateral view large and nearly circular in shape (Thomson and Armitage 2018: fig. 6A)	***N.atopa* Thomson & Armitage, 2018**
–	Bracteole in lateral view thin over length, not nearly circular (Fig. 2A; Keth et al. 2015: fig. 42A)	**39**
39	Inferior appendage in ventral view terminating in elongate spines (Keth et al. 2015: fig. 76C) or short spines (Keth et al. 2015: fig. 42C)	**40**
–	Inferior appendage in ventral view not terminating in elongate or short spines (Fig. 2B)	***N.abrebotella* sp. nov.**
40	Inferior appendage in ventral view terminating in elongate spines (fig. 76C in Keth et al. 2015); phallus with elongate lateral rod apically (Keth et al. 2015: fig. 76B)	***N.armata* Botosaneanu, 1993 (in Botosaneanu and Alkins-Koo 1993)**
–	Inferior appendages in ventral view terminating in short spines (Keth et al. 2015: fig. 42C); phallus without elongate lateral rod apically (Keth et al. 2015: fig. 42B)	***N.amplector* Keth, 2004**
41	Phallus terminating in a spine (Harris and Armitage 2019: fig. 14D) or spines (Harris 1990: fig. 8D; Keth et al. 2015: fig. 53B)	**42**
–	Phallus not terminating in a spine or spines (Fig. 16D; Keth et al. 2015: fig. 53B)	**44**
42	Phallus terminating in a single spine (Harris and Armitage 2019: fig. 14D); subgenital plate in lateral view with complex terminal rod projecting ventrad beyond inferior appendage and subterminal rod serrate at tip (Harris and Armitage 2019: fig. 14A)	***N.rambala* Harris & Armitage, 2019**
–	Phallus terminating in a pair of rods which can be fine (Harris 1990: fig. 8D), or stout (Keth et al. 2015: fig. 53B); subgenital plate various but not complex (Harris 1990: fig. 8A; Keth et al. 2015: fig. 53A)	**43**
43	Rods at apex of phallus stout and elongate (Keth et al. 2015: fig. 53B); inferior appendages not bifid laterally, but triangular in ventral view (Keth et al. 2015: fig. 53A–C)	***N.tuxtla* Bueno-Soria, 1999**
–	Rods at apex of phallus thin and short (Harris 1990: fig. 8D); inferior appendages bifid laterally and elongate ventrally (Harris 1990: fig. 8A, B)	***N.flowersi* Harris, 1990**
44	Inferior appendages short and triangular in lateral view (Fig. 16A) and ventral view (Fig. 16B)	***N.veraguasensis* sp. nov.**
–	Inferior appendages elongate and rectangular in lateral and ventral views (Oláh and Johanson 2011: figs 144, 146)	***N.kampa* Oláh & Johanson, 2011**

## ﻿Discussion

With the publication of this paper, there are now 226 species of *Neotrichia* which occur in North, Central, and South America and the West Indies (Armitage et al. 2022; Gama-Neto and Passos 2023; Harris et al. 2023; Thomson 2023). Of these, 31 species are known from Mexico and 50 species from Central America, with Panama’s 45 species comprising the majority of the latter. We suspect that many of Panama’s 33 endemic species of *Neotrichia* would lose that status once the Trichoptera fauna in the other Central American countries becomes better known. Other than Belize, with five endemic (and total) species, no other Central American country hosts any endemics. Three species are recorded from Nicaragua, one from Costa Rica, and none are recorded from El Salvador, Guatemala, and Honduras (Thomson 2023). Given the close relationship between the faunas of Costa Rica and Panama, we expect that they share many species. In South America, Brazil is the dominant country for this genus with 48 recorded species (Gama-Neto and Passos 2023; Santos 2023), and has the majority of the endemic species. It shares only two species with Panama (*N.tauricornis* Malicky, 1980; *N.parabullata* Harris & Armitage, 2015), so that the faunas of these two countries, though currently similar in size, are more or less distinct.

Given the rapid increase in *Neotrichia* species we have found in Panama during the last eight years (42 species, with ~ 75% of them new to science), we are no longer surprised, but still delighted, at what is revealed in our samples. With most of Panama as yet unexplored, the possibility of 75, or even 100, species of this genus in Panama is not beyond the imagination. Whereas we are just now beginning to see second and third locations for some of the *Neotrichia* we have previously described, “new” species remain the central theme of our results.

It is well known that keys to species, while useful, are usually out of date as soon as they are written. However, we developed the key presented above out of self-defense to help manage the current list, to provide a framework for new keys in the future, and, most of all, to assist us in identifying and verifying new species as they appear. In Keth et al. (2015) an attempt was made to “further organize” primarily Nearctic species into species groups. However, some of the new *Neotrichia* species we are finding in Panama raise some doubts about the validity of those groupings. Increasing knowledge of the sampling universe vis-à-vis *Neotrichia* has not necessarily brought clarification in this regard, as might be expected. Other types of analyses, including molecular, need to be brought to bear to supplement what we think we know about this most interesting genus.

## Supplementary Material

XML Treatment for Neotrichia

XML Treatment for Neotrichia abrebotella

XML Treatment for Neotrichia candela

XML Treatment for Neotrichia codaza

XML Treatment for Neotrichia embera

XML Treatment for Neotrichia flennikeni

XML Treatment for Neotrichia honda

XML Treatment for Neotrichia landisae

XML Treatment for Neotrichia lenati

XML Treatment for Neotrichia mindyae

XML Treatment for Neotrichia panamensis

XML Treatment for Neotrichia parajarochita

XML Treatment for Neotrichia paraxicana

XML Treatment for Neotrichia snixae

XML Treatment for Neotrichia spangleri

XML Treatment for Neotrichia veraguasensis

XML Treatment for Neotrichia minutisimella

XML Treatment for Neotrichia vibrans

## ﻿Additional information

### Author ORCIDs

Steven C. Harris https://orcid.org/0000-0002-6432-7462

Brian J. Armitage https://orcid.org/0000-0003-3182-1533

Tomás A. Ríos González https://orcid.org/0000-0003-0590-6488

### Data availability

All of the data that support the findings of this study are available in the main text.
